# Decreased plasma phospholipid concentrations and increased acid sphingomyelinase activity are accurate biomarkers for community-acquired pneumonia

**DOI:** 10.1186/s12967-019-2112-z

**Published:** 2019-11-11

**Authors:** Haroon Arshad, Juan Carlos López Alfonso, Raimo Franke, Katina Michaelis, Leonardo Araujo, Aamna Habib, Yuliya Zboromyrska, Eva Lücke, Emilia Strungaru, Manas K. Akmatov, Haralampos Hatzikirou, Michael Meyer-Hermann, Astrid Petersmann, Matthias Nauck, Mark Brönstrup, Ursula Bilitewski, Laurent Abel, Jorg Sievers, Jordi Vila, Thomas Illig, Jens Schreiber, Frank Pessler

**Affiliations:** 1 Research Group “Biomarkers for Infectious Diseases”, TWINCORE Centre for Experimental and Clinical Infection Research, Feodor-Lynen-Str. 7, 30625 Hannover, Germany; 2 Helmholtz Centre for Infection Research, Brunswick, Germany; 3 Centre for Individualised Infection Medicine, Hannover, Germany; 4 Clinic for Pneumology, Otto-von-Guericke University, Magdeburg, Germany; 5 Department of Chemical Biology, Helmholtz Centre for Infection Research and German Center for Infection Research (DZIF), Brunswick, Germany; 6 Department of Clinical Microbiology, Biomedical Diagnostic Centre (CDB), Hospital Clinic, School of Medicine, University of Barcelona, Institute of Global Health (ISGlobal), Barcelona, Spain; 7 Laboratory of Human Genetics of Infectious Diseases, Necker Branch, INSERM, Paris, France; 8 Paris Descartes University, Imagine Institute, Paris, France; 9 Giles Laboratory of Human Genetics of Infectious Diseases, Rockefeller Branch, Rockefeller University, New York, USA; 10 Hannover Unified Biobank, Hannover Medical School, Hannover, Germany; 11 Department of Systems Immunology and Braunschweig Integrated Centre of Systems Biology, Helmholtz Centre for Infection Research, Brunswick, Germany; 12 Institute for Clinical Chemistry and Laboratory Medicine, University Medicine Greifswald, Greifswald, Germany; 13 Clinical Microbiology, GlaxoSmithKline, Collegeville, PA USA; 14 Present Address: Clinical Development, ViiV Healthcare, Brentford, UK; 15 DZHK (German Centre for Cardiovascular Research), Partner Site Greifswald, University Medicine, Greifswald, Germany; 16 Present Address: UMG-Laboratory, University Medicine Göttingen, Göttingen, Germany

**Keywords:** Biomarkers, Chronic obstructive pulmonary disease, Community-acquired pneumonia, Glycerophospholipids, Infection, Lipidomics, Lung disease, Mass spectrometry, Metabolism, Metabolomics, Sphingomyelinase

## Abstract

**Background:**

There continues to be a great need for better biomarkers and host-directed treatment targets for community-acquired pneumonia (CAP). Alterations in phospholipid metabolism may constitute a source of small molecule biomarkers for acute infections including CAP. Evidence from animal models of pulmonary infections and sepsis suggests that inhibiting acid sphingomyelinase (which releases ceramides from sphingomyelins) may reduce end-organ damage.

**Methods:**

We measured concentrations of 105 phospholipids, 40 acylcarnitines, and 4 ceramides, as well as acid sphingomyelinase activity, in plasma from patients with CAP (n = 29, sampled on admission and 4 subsequent time points), chronic obstructive pulmonary disease exacerbation with infection (COPD, n = 13) as a clinically important disease control, and 33 age- and sex-matched controls.

**Results:**

Phospholipid concentrations were greatly decreased in CAP and normalized along clinical improvement. Greatest changes were seen in phosphatidylcholines, followed by lysophosphatidylcholines, sphingomyelins and ceramides (three of which were upregulated), and were least in acylcarnitines. Changes in COPD were less pronounced, but also differed qualitatively, e.g. by increases in selected sphingomyelins. We identified highly accurate biomarkers for CAP (AUC ≤ 0.97) and COPD (AUC ≤ 0.93) vs. Controls, and moderately accurate biomarkers for CAP vs. COPD (AUC ≤ 0.83), all of which were phospholipids. Phosphatidylcholines, lysophosphatidylcholines, and sphingomyelins were also markedly decreased in *S. aureus*-infected human A549 and differentiated THP1 cells. Correlations with C-reactive protein and procalcitonin were predominantly negative but only of mild-to-moderate extent, suggesting that these markers reflect more than merely inflammation. Consistent with the increased ceramide concentrations, increased acid sphingomyelinase activity accurately distinguished CAP (fold change = 2.8, AUC = 0.94) and COPD (1.75, 0.88) from Controls and normalized with clinical resolution.

**Conclusions:**

The results underscore the high potential of plasma phospholipids as biomarkers for CAP, begin to reveal differences in lipid dysregulation between CAP and infection-associated COPD exacerbation, and suggest that the decreases in plasma concentrations are at least partially determined by changes in host target cells. Furthermore, they provide validation in clinical blood samples of acid sphingomyelinase as a potential treatment target to improve clinical outcome of CAP.

## Background

Community-acquired pneumonia (CAP) is the sixth most common cause of death worldwide and is a major burden on healthcare resources [1]. According to recent reports, the overall annual incidence of CAP in Europe is 1.2 per 1000 persons and it increases with age to 14 per 1000 person a year in adults aged ≥ 65 years [2]. There have been intense efforts to identify host biomarkers for CAP [3, 4]. These studies have been geared predominantly toward identifying biomarkers to [1] aid in early diagnosis, for instance to distinguish between viral and bacterial etiologies to improve antibiotic stewardship, [2] determine more accurately the required duration of therapy, and [3] improve risk stratification and prediction of clinical outcome. Another important goal of biomarker profiling of patient biosamples is to study pathogenesis, e.g. by elucidating molecular pathways that are strongly associated with a disease state and may constitute targets for patient-directed adjunct treatments to improve outcome, for instance by reducing end-organ damage.

Lipids are involved in a variety of processes intimately connected with the pathogenesis of pneumonia and related infectious diseases. For instance, membrane lipids are crucial players in intracellular signaling events that are found across immune cells and resident lung cells, e.g. signaling due to phospholipases which release bioactive molecules such as fatty acids (e.g., arachidonic acid), sphingolipids and lysophosphatidylcholines (lysoPC) and their derivatives [5]. Cholesterol, sphingomyelins (SM) and phosphatidylcholines (PC) are involved in the formation of the immunological synapse, and lipids play important roles in macrophage activation [6], NK cell function 2011 [7], and differentiation and activity of T and B effector cells [8, 9]. Lipid-mediated signaling has also been shown to be involved in regulating processes intimately associated with inflammation- and infection-associated end-organ and cell damage, notably apoptosis, autophagy [10], and pulmonary fibrosis [11]. In particular, ceramides are considered to be proinflammatory lipids in lung epithelium, and pharmacological inhibition of their synthesis by acid sphingomyelinase may reduce organ damage due to reactive oxygen species and inflammation in mouse models of sepsis [12, 13] and *Pseudomonas aeruginosa* infection in cystic fibrosis [14]. Moreover, intracellular oxidation of saturated lipids and ceramides by reactive oxygen species leads to the synthesis of peroxides and induces ferroptosis [15]. Considering these and other pieces of evidence, it has recently been suggested that acid sphingomyelinase could serve as a treatment target not just for cystic fibrosis [16] but for a range of bacterial infections [17]. Acylcarnitines (AC) are fatty acids covalently bound to carnitine, which are imported into mitochondria where the fatty acid is used for energy generation via beta-oxidation. Increased AC concentrations in plasma have been associated with organ damage and mortality in sepsis [18], suggesting that they may play similar roles in CAP as well. Lastly, significantly decreased concentrations of lysoPC have been found in CAP [19, 20].

Considering these intimate connections between lipids and processes relating to the pathogenesis of pneumonia, it is conceivable that changes in plasma lipid populations can be mined for biomarker discovery. In addition, identification of disease-associated metabolites and their biosynthetic pathways could potentially lead to the development of adjunct, host-directed treatments aiming to reduce end-organ damage and to improve clinical outcome. We have therefore applied a targeted lipidomic screen and an assay for acid sphingomyelinase activity to plasma samples from (1) patients with CAP during acute presentation and convalescence, (2) COPD exacerbation with suspected infection as a clinically potentially similar disease of different etiology (“disease control”), and (3) age- and sex-matched control subjects, and aimed to (1) assess global changes in plasma lipid populations and identify specific biomarkers for a diagnosis of CAP (also in contrast to COPD exacerbation) and for its clinical resolution and (2) test whether increased acid sphingomyalinase activity in peripheral blood is a feature of these disorders. In addition, we used a cellular model of *S. aureus* infection to test whether any observed changes in plasma phospholipid concentrations might originate at the level of infected or inflammation-exposed host cells.

## Methods, materials and participants

### Study design and study population

This was a prospective, hospital-based study conducted at Otto-von-Guericke-University Hospital (Magdeburg, Germany) and Hospital Clinic (Barcelona, Spain) between March 2014 and November 2015. We recruited patients (age ≥ 18 years) with CAP (n = 29) or COPD exacerbation with suspected infection (n = 13), both requiring hospitalization, and an age- and sex-matched control group [n = 33, consisting of structural or degenerative orthopedic disorders (n = 28) and healthy volunteers (n = 5)] without systemic inflammation as evidenced by clinical and laboratory findings. All participants provided written informed consent. The study was conducted according to the Helsinki Declaration and was approved by the respective Ethics Committees (Otto-von-Guericke University Magdeburg: file no. 36/14; Hospital Clinic Barcelona: file no. HCB 2013/8396). Inclusion criteria for CAP were (1) evidence of pulmonary infiltrate on chest X-ray, (2) presence of at least two of the following four symptoms: cough, purulent sputum production, dyspnea, pleuritic chest pain, (3) at least two vital sign abnormalities out of fever/hypothermia, hypotension, tachycardia, tachypnea, (4) one out of hypoxemia, evidence of pulmonary consolidation, leukocytosis or leukopenia, and (5) need for inpatient treatment as judged by the examining physician. Inclusion criteria for COPD exacerbation with suspected infection were (1) documented history of COPD, (2) sudden deterioration in respiratory status/lung function, and (3) symptoms (increase in sputum production, cough) and/or laboratory abnormalities (increased CRP and/or detection of a respiratory pathogen) suggestive of an infectious trigger or coexisting airway infection. Exclusion criteria were a diagnosis of cancer or a chronic inflammatory disease (e.g., rheumatoid arthritis). Co-existing pneumonia was not an exclusion criterion for COPD, but it was not detected in any of the 13 participants in this group. At baseline (day (d)1), the following parameters were assessed in CAP and COPD: medical history, lung function tests, chest radiograph, vital signs (heart rate, blood pressure and body temperature), complete blood count, liver and kidney chemistry, and blood gas analysis. Furthermore, clinical data and blood samples were collected on d 2 and 4, and at two follow-up visits (1–14 days (f1) and 4–6 weeks (f2) after completion of antibiotic treatment). Nasal/pharyngeal swabs, sputum, urine and additional blood samples were collected and subjected to microbiological analysis as clinically indicated. Blood samples from Controls were obtained once. All biosamples were collected and stored according to international biobanking standards (DIN EN ISO 9001:2008) and unified SOPs were used for all samples. Plasma C-reactive protein (CRP) and procalcitonin (PCT) levels were measured in all samples in a single batch after conclusion of recruitment. CRP was measured nephelometrically on the Dimension VISTA 1500 platform, using commercially available reagents and calibrators (Siemens Healthcare GmbH, Eschborn, Germany). The PCT assay was purchased from Thermo Fisher (Dreieich, Germany) and performed on the Centaur XPT immunochemistry analyzer (Siemens Healthcare GmbH, Eschborn, Germany).

### Lipidomic profiling

Concentrations of 145 lipids (76 PC, 14 lysoPC, 15 SM, and 40 AC) were measured with the AbsoluteIDQ^®^ p180 kit (Biocrates, Life Science AG, Innsbruck, Austria) by direct flow injection analysis using a triple-quadrupole mass spectrometer (AB Sciex, QTRAP^®^ 6500). Extraction of metabolites and all analytical assays were performed according to the manufacturer’s recommendations (UM_p180_ABSciex_11 and Application Note 1003-1, Biocrates Life Science AG, Innsbruck, Austria). Peak integration and calculation of metabolite concentrations were performed with the MetIDQ™ software tool (Biocrates Life Science AG, Innsbruck, Austria). The following nomenclature was used: sphingomyelin = SM; hydroxysphingomyelin = SM (OH); phosphatidylcholine = PC; aa = both side chains are fatty acids linked to a glycerol backbone by ester bonds; ae = one of the side chains is a fatty alcohol linked to the glycerol backbone by an ether bond; Cx:y; x = total number of carbon atoms and y = total number of double bonds in the side chains. In order to investigate the extent of reprogramming across classes of lipid molecules or selected enzyme activities, the MetIDQ™ Ratio Explorer software tool (Biocrates Life Sciences, [21]) was used to define 47 “metabolic indicators” (Additional file 1: Table S1), i.e. the sums of mean concentrations of biochemically related lipids (lipid classes, n = 11), ratios involving one or two lipid classes (n = 11), and ratios of specific lipid pairs (n = 25, 23 of which were custom-added to the Explorer for this study).

### Targeted assays for ceramides

For the analysis shown in Fig. 6a–c, a liquid chromatography (LC) coupled triple-quadrupole time-of-flight mass spectrometer (QTOF5600, Sciex) was used to quantify selected ceramides. The ceramide standards C16:0 (860516), C18:0 (860518), C24:0 (860524) and C24:1 (860525) were purchased from Avanti polar lipids (Alabama, US). Gradient-grade organic solvents and other chemicals included chloroform, methanol, and water (9175, 9017, 8402, 4218, J.T. Baker), formic acid, ammonium acetate, ammonium formate, dimethyl sulfoxide (06473, 55674, 70221, 472301 respectively, Sigma), and bovine serum albumin (BSA, 15269, ICN Biomedicals). A solution of pure analytes was injected into the machine for identification of individual masses and to obtain a chromatogram. The optimized MS settings were: gas 1: 60 psi, gas 2: 75 psi, curtain gas: 45 psi, ion source temperature: 400 °C, ion spray voltage floating: 4500 V and mass range: 50–1000 Da. An acquisition method was established in positive ion mode to ensure the accurate identification of each analyte. The optimized HPLC parameters included: mobile phase; A, methanol/water (3/1, v/v, 1% formic acid, 20 mM ammonium formate); B, methanol/formic acid (99/1, v/v, 20 mM ammonium formate), column oven temperature 60 °C; mobile phase gradient: initial mobile phase was 80% B and the gradient was applied to attain 100% B within the first 10 min and the column was re-equilibrated for the last 3 min and flow rate of 0.7 ml/min. Metabolites were extracted using the classical Bligh and Dyer extraction protocol [22] with SM 17:0 as internal standard (ISTD). To assess performance of this extraction protocol, plasma was spiked with ISTD in five replicates and extraction was performed, followed by measurement of ISTD peak areas. A calibration curve in the concentration range from 0.02 to 10 µM with an ISTD was prepared in BSA and injected into the LC–MS/MS system to estimate limit of detection (LOD) and lower limit of quantification.

### Acid sphingomyelinase enzyme assay

Enzymatic activity in plasma samples was measured using the Amplex^®^ Red Sphingomyelinase Assay Kit (Invitrogen) according to the manufacturer’s instructions. This assay is based on signal detection from a fluorescent product (resorufin) which is generated as an end product of the sphingomyelin cleavage reaction.

### Infection of human cells with *S. aureus*

The myelomonocytic cell line THP1 was propagated in RPMI 1640 medium (GIBCO^®^ Life Technologies™) supplemented with 10% fetal bovine serum (FBS) and 2 mM l-glutamine. To obtain differentiated cells (dTHP1), 5 × 10^6^ THP1 cells/flask (T-75) in 25 ml of complete RPMI media were differentiated with 200 nM phorbol-12-myristate-13-acetate (PMA, Sigma-Aldrich, product no. P-8139) for 48 h and then incubated in fresh RPMI medium for another 24 h. 5 × 10^6^ A549 (adenocarcinoma) cells were propagated in 25 ml complete DMEM medium supplemented with 10% FCS and 2 mM l-glutamine, and were allowed to settle for 24 h. Cells were infected with GFP-expressing *S. aureus* strain SH1000 at an MOI of 25 and incubated at 37 °C for 2 h. Cells were subsequently washed twice with PBS, and non-ingested bacteria were inactivated by adding gentamicin (100 µg/ml) to the post-infection medium. Accutase solution was used to detach the A549 cells, whereas dTHP1 cells were detached with a cell scraper. Cells were then centrifuged to make a cell pellet, which was stored at − 80 °C. Frozen cell pellets were thawed on ice. 250 µl ice cold extraction solvent (ethanol/0.01 M phosphate buffer with 85/15, V/V) was used to re-suspend the cell pellet, which was then sonicated in an ice bath for 3 min. Liquid nitrogen was used for snap freezing. The sonicating/freezing/sonicating cycles were repeated 3 times. The samples were then centrifuged for 5 min with 20,817*g* at 4 °C and lipid concentrations in the supernatant analyzed using the absolute*IDQ*^®^ p180 kit as described above for plasma.

### Reanalysis of published gene expression data sets

Data sets from two studies featuring mRNA expression in CAP [23, 24] were accessed through GEO under accession numbers GSE40012 and 42830, respectively. Normalized expression values for the two isoforms of sphingomyelin phosphodiesterase 1 (*SMPD1*, OMIM: 607608; also known as acid sphingomyelinase) were obtained using the GEO2R web tool [25] and significance of between-group expression differences assessed with the Mann–Whitney U test.

### Statistical analysis

We included only those analytes with measured values above LOD of the AbsoluteIDQ^®^ p180 kit (as reported by the manufacturer) in at least 75% of the samples. Missing values were then replaced by imputation using linear regression models based on age, sex, disease state, and sample collection time point. The Mann–Whitney U test and Kruskal–Wallis H-test were used to assess significance of differences between groups, with significance defined as a p value of < 0.05. The Benjamini–Hochberg correction was implemented to correct for multiple testing using a false discovery rate (FDR) equal to 0.05. Statistical tests were performed using the open-source stats module of the Python’s SciPy statistic library (https://docs.scipy.org/doc/scipy/reference/stats.html) and the *MNE*-Python software package v0.18.1 (http://mne-tools.github.io/dev/index.html). For principal component analysis (PCA), data were standardized by removing the mean and scaling to unit variance and then analyzed using the *scikit*-*learn Python* library for machine learning (https://scikit-learn.org). Receiver operating characteristic (ROC) curves were computed by means of the function roc_curve() of the sklearn.metrics module, using the logistic regression classifier in the sklearn.linear_model module with a stratified k-fold cross-validation (k = 5) repeated 25 times to estimate the mean area under the curve (AUC) and confidence intervals (CI). We defined biomarker candidates (for the sake of simplicity in the text referred to as biomarkers) as analytes with an AUC ≥ 0.80 (“excellent classification” according to Hosmer and Lemeshow [26]), an asymptotic p value of < 0.05 (Mann–Whitney U test), and 95% confidence intervals (CI) not crossing the chance-line of AUC = 0.50. For linear fitting slope (LFS) analysis, the ratios of medians of each time-observation versus control were fitted to a linear function using the function polyfit() of the NumPy package for scientific computing (https://www.numpy.org). Then, the distance of each ratio of medians to the fitted line was divided by the difference between the maximum and minimum ratio of medians, and the sum of the resulting values was then normalized by the number of observations (equal to 5), which is referred to as the normalized distance to fitting (NDF). Pearson correlation coefficients were obtained with the function pearsonr() of the *scipy*.stats module and used to assess correlations between metabolites and CRP and PCT. To assess the effect of sample size on biomarker discovery, increasing the size of the COPD group to that of CAP was modeled using the function RandomOverSampler() in the imbalanced-learn package (https://imbalanced-learn.readthedocs.io/en/stable/), which over-samples the minority (COPD) class by picking samples at random with replacement.

## Results

### Study population

Table 1 summarizes sociodemographic and selected clinical characteristics of the three study groups. There were no significant differences in age and sex among CAP, COPD, and Controls. All CAP and COPD patients received antimicrobial treatment either before hospital admission or were started on an antibiotic upon admission. Consistent with standard treatment, substantially more COPD than CAP patients were started on systemic corticosteroid treatment upon admission. Approximately 50% each of CAP patients had low (Pneumonia Severity Index [PSI], I–III) or moderate-to-high disease severity (PSI, IV–V, three of whom developed sepsis). One CAP patient was lost to follow-up after d4, but all 28 patients who returned for the f1 and/or f2 visits were in clinical remission at that time. Completeness of study visits and blood samples in CAP is summarized in Additional file 1: Table S2. Disease activity in COPD (GOLD grade) was weighted more toward severe disease. Consistent with clinical experience, likely causative pathogens were detected in only about half of the cases. As expected, CRP and PCT concentrations were highest in CAP, intermediate in COPD, and normal in Controls. Thus, the CAP and COPD groups had the major clinical characteristics expected from the natural history of these disorders, but CAP also included several mild cases who were admitted to hospital according to the clinical judgment of the admitting or referring physician.

**Table 1:** Demographic, clinical and laboratory characteristics of the study population

	CAP (n = 29)	Infection-associated COPD exacerbation (n = 13)	Controls (n = 33)	p value	
				All groups^a^	CAP vs. COPD^b^
Demographics					
Sex					
Female (%)	38	46	36	0.30	0.25
Male (%)	62	54	64		
Median age (range)	60 (24–90)	62 (55–81)	59 (24–90)	0.78	0.31
Medical history (past or current)					
Diabetes (%)	38	17	24	2.0 × 10^−03^	9.0 × 10^−04^
Cardiovascular disease (%)	28	50	24	1.6 × 10^−04^	1.4 × 10^−03^
Cancer (%)	10	30	6	< 1.0 × 10^−05^	4.1 × 10^−04^
Treatment (at time of first blood sample)					
Antimicrobial (%)	100	100	0	NA	NA
Corticosteroid (%)	22	62	3.3	< 1.0 × 10^−05^	< 1.0 × 10^−05^
Disease severity					
PSI risk class (%)					
I (low)	21	NA	NA	NA	NA
II (low)	20	NA	NA	NA	NA
III (low)	14	NA	NA	NA	NA
IV (moderate)	24	NA	NA	NA	NA
V (high)	21	NA	NA	NA	NA
GOLD grades (%)					
I (mild)	NA	0	NA	NA	NA
II (moderate)	NA	25	NA	NA	NA
III (severe)	NA	25	NA	NA	NA
IV (very severe)	NA	50	NA	NA	NA
Laboratory results					
CRP (mg/l, ref. < 5 mg/l)	102 (3.1–428)	14 (3.1–91)	3.1 (3.1–7.1)	2.5 × 10^−12^	2.5 × 10^−05^
PCT (ng/l, ref. < 0.5 ng/l)	0.23 (0.02–38)	0.09 (0.02–1.0)	0.02 (0.02–0.08)	5.0 × 10^−09^	7.1 × 10^−03^
Pathogen detected (%)	41	54	NA	NA	NA

*CRP* C-reactive protein, *GOLD* Global Initiative for Chronic Obstructive Lung Disease, *NA* not applicable, *PCT* procalcitonin, *PSI* Pneumonia Severity Index

^a^Kruskall–Wallis test

^b^Mann–Whitney U test (continuous variables), Chi^2^ test (categorical variables)

### Metabolite detection across all samples

Of the 145 potentially detectable lipid analytes, measured concentrations of 111 (77%) were above LOD in ≥ 75% of the samples and were included in the subsequent analyses (Additional file 1: Fig. S1A). Analyte detection was much more efficient for glycerophospholipids and sphingolipids than for acylcarnitines (Additional file 1: Fig. S1B). These results agreed well with information provided by the manufacturer of the MetIDQ™ p180 kit and published data [27, 28].

### Changes in lipid populations are more extensive in CAP than in COPD and normalize with clinical recovery

**Fig. 1:**
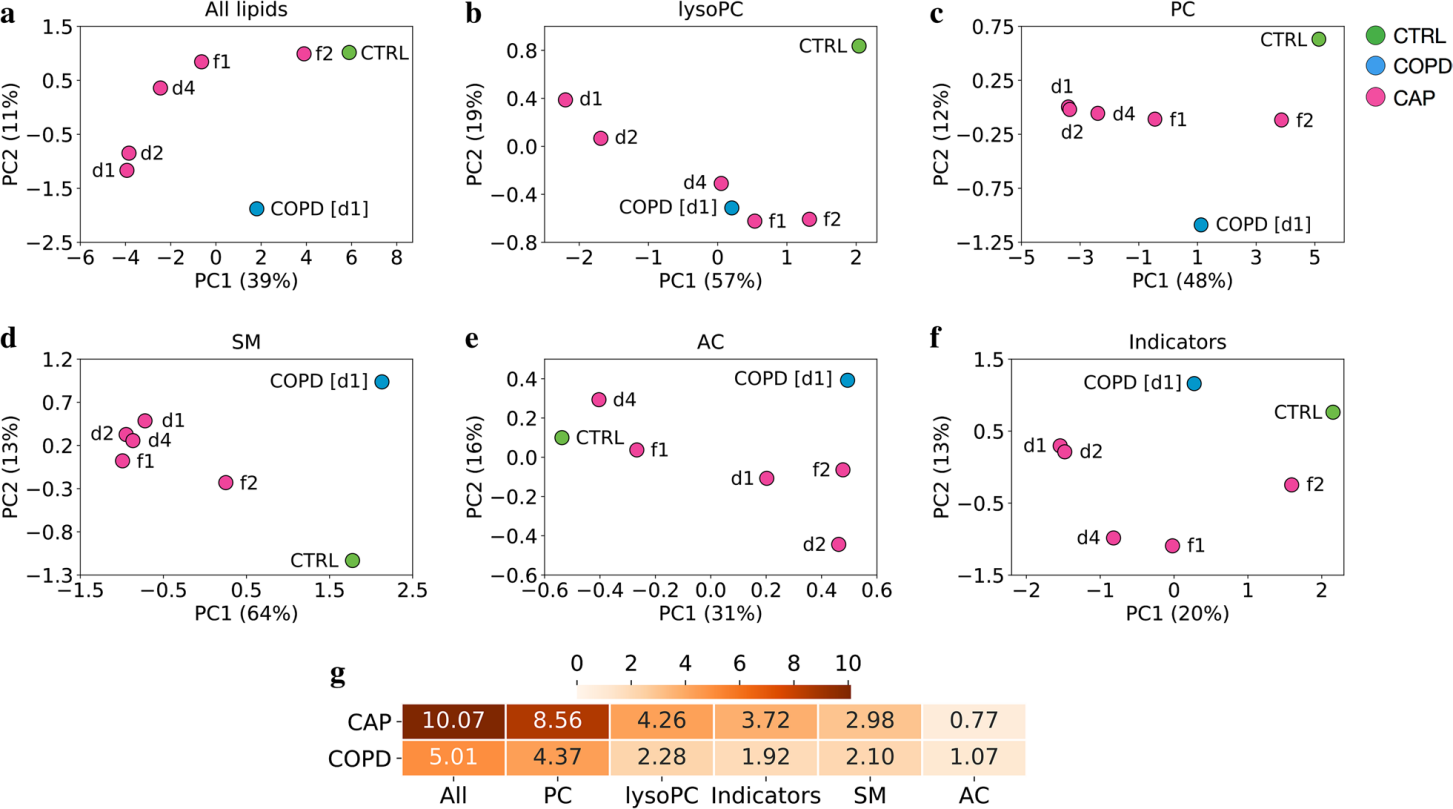
Plasma lipid reprogramming is greater in CAP than COPD and normalizes in CAP with treatment. Principal component analysis (PCA) based either on concentrations of the 111 lipid analytes that passed the quality screen shown in this figure or on the 47 metabolic indicators. Each circle represents the centroid of all samples in the respective group/time point. **a** Analysis based on concentrations of all 111 lipid analytes; **b** lysoPC only; **c** PC only; **d** SM only; **e** AC only; **f** metabolic indicators. **g** Mean Euclidian distances on day 1 between CAP or COPD and Controls, based on all lipid analytes, each subclass, or on the metabolic indicators. Metabolites: AC, acylcarnitines; lysoPC, lysophosphatidylcholines; PC, phosphatidylcholines; SM, sphingomyelins

Principal component analysis (PCA) was then used to test whether differences in the lipid profiles reflected the relationships expected from the clinical diagnoses, as well as clinical recovery in CAP (Fig. 1; the contribution of each lipid to the first and second principle components is shown in Additional file 1: Fig. S2). The CAP baseline and COPD groups (d1) clustered distinctly away from each other and from Controls, and analysis of CAP at subsequent time points revealed a “normalization” of lipid populations in CAP in that they clustered progressively closer to Controls (Fig. 1a). Similar patterns were observed when profiles of the subclasses lysoPC (Fig. 1b), PC (Fig. 1c), and SM (Fig. 1d) were analyzed, whereas the relationships based on AC did not follow a discernable pattern (Fig. 1e). Analysis of the metabolic indicator patterns essentially confirmed the results based on the individual metabolites (compare Fig. 1f and a). The overall changes in lipid concentrations at baseline (as measured by Euclidian distance to Controls) were markedly greater in CAP than in COPD, and among the subgroups it was greatest in PC and lowest in AC (Fig. 1g). These results suggested a striking, higher degree of plasma lipid “reprogramming” in CAP than in COPD, which agreed well with the much higher degree of systemic inflammation (CRP, PCT) in CAP (Table 1).

### Marked decrease in membrane phospholipids in CAP plasma

**Fig. 2:**
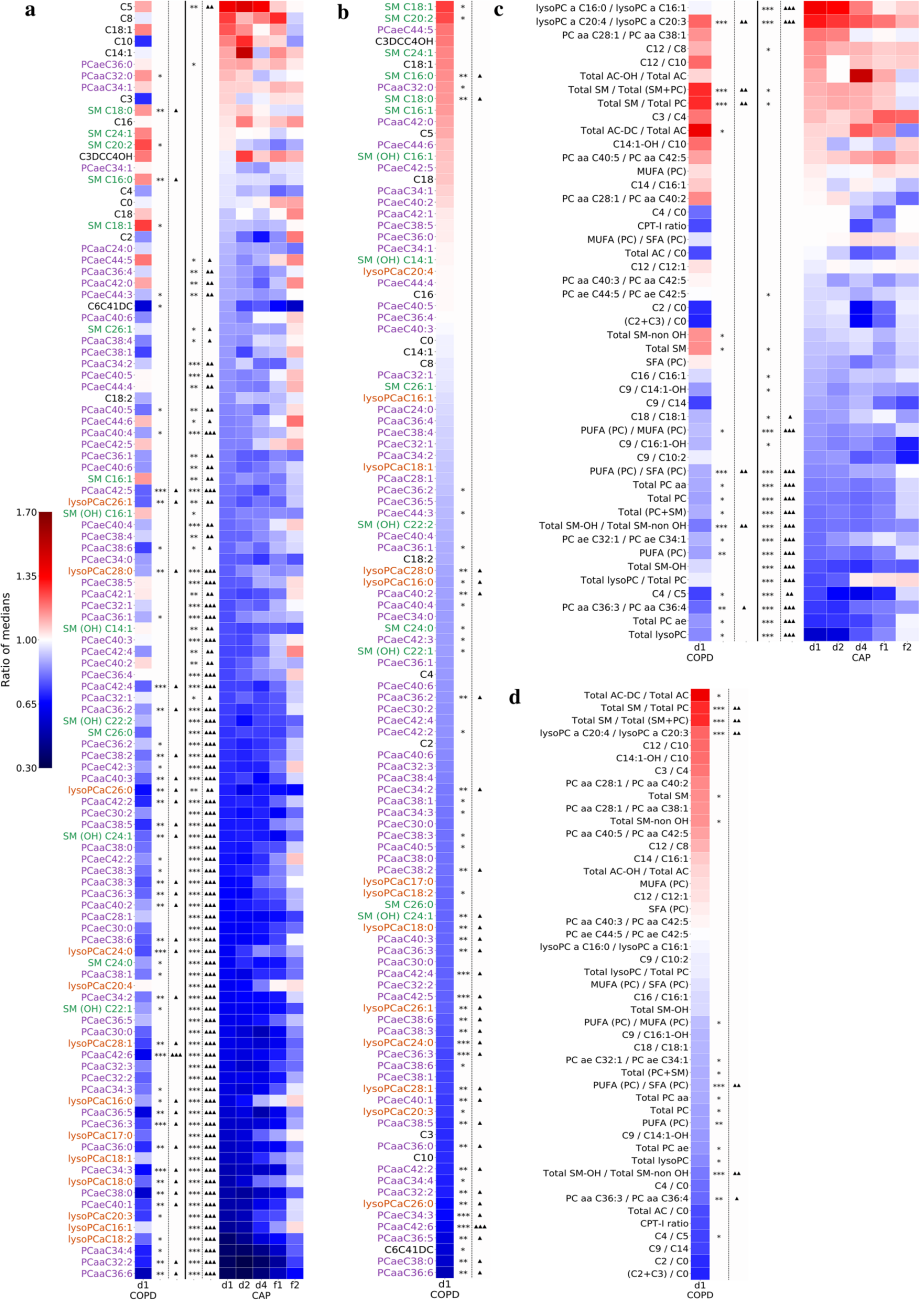
Massive decrease in phospholipid concentrations governs changes in plasma lipids in CAP. **a** Analysis based on the 111 lipid analytes and 47 metabolic indicators. Data from COPD samples are displayed in the left column and CAP data in the right column. The lipid analytes are arranged in descending order according to ratio of median concentrations CAP d1/Controls (“fold change”). A major drop in membrane phospholipid concentrations is evident in CAP. **b** COPD only, same data as used in A, but showing differential expression in COPD, arranged in descending order according to fold change COPD vs Controls. **c** Analysis based on the 47 metabolic indicators. The indicators are ranked vertically according to descending ratio of median values in CAP d1/Controls (C). **d** COPD only, same data as used in C, but showing differential expression in COPD, arranged in descending order according to fold change COPD vs Controls. Differential expression (fold change) is expressed on a linear scale by the color scheme in the legend shown in **a**. Lipid classes are identified by the font colors identified in the legend. *p < 0.05, **p < 0.01, ***p < 0.001 (Mann–Whitney U test). ^Δ^p < 0.05, ^ΔΔ^p < 0.01, ^ΔΔΔ^p < 0.001 (FDR-corrected). Metabolite: AC, acylcarnitines; lysoPC, lysophosphatidylcholines; PC, phosphatidylcholines; SM, sphingomyelins. A comprehensive lipid nomenclature is detailed in “Methods, materials and participants”

Concentrations of the majority of lipid analytes (85/111 = 77%) were significantly changed in CAP baseline (d1) with respect to Controls, most of which (83/85 = 98%) were downregulated, and all downregulated analytes were membrane lipids, i.e. PC, lysoPC or SM (Fig. 2a). Only one AC (1/10 = 10%; C5) was differentially abundant in CAP at baseline, but also (together with PCaeC36:0) constituted one of the only two upregulated analytes. Consistent with the PCA shown in Fig. 1, lipid concentrations in CAP trended to “normalize” across the time course towards concentrations measured in Controls, but a substantial fraction of analytes did not normalize completely. Also, there were some clear differences in the direction of concentration change (up vs. down) between COPD and CAP (Fig. 2a, b). Similar results were obtained with the metabolic indicators (Fig. 2c, d), which also revealed significantly elevated ratios of lysoPCaC16:0/C16:1 and lysoPCaC20:4/C20:3, likely indicating differences in conversion of the parent PCs by phospholipase A2, as direct (de)saturases that modify PC-bound fatty acids are not known. This analysis of metabolic indicators also revealed that changes in AC metabolism were somewhat more pronounced than suggested by abundance data of individual analytes, as 5/16 (31%) AC indicators were differentially regulated in CAP at baseline compared to Controls (Fig. 2c). In addition, a tendency toward changes in fatty acid oxidation for cell respiration was seen in COPD, as exemplified by an increased ratio of dicarboxylacylcarnitines/total acylcarnitines indicating increased utilization of ω-oxidation (a salvage pathway when β-oxidation is impaired) for ATP synthesis (Fig. 4f).

**Fig. 3:**
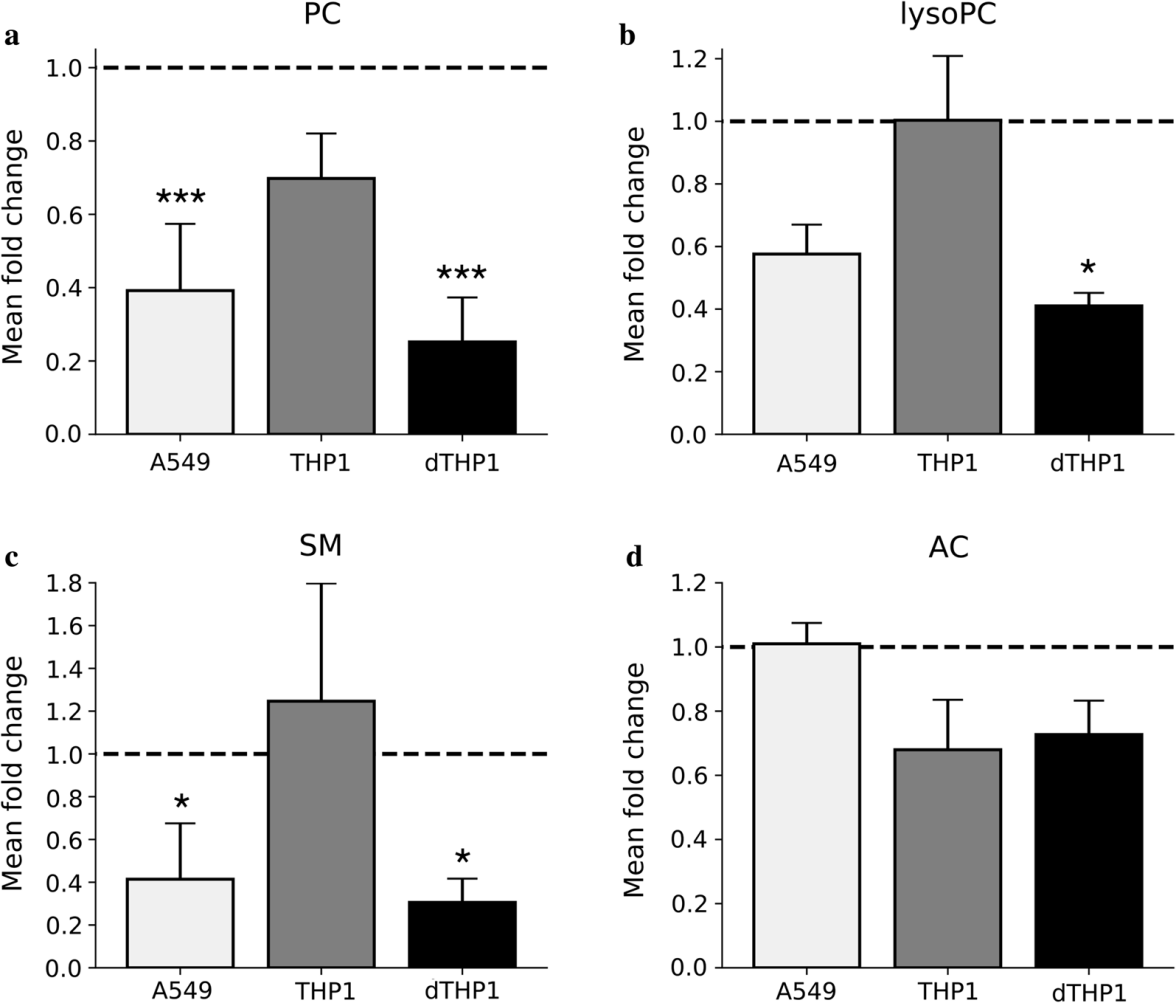
Infection with *S. aureus* leads to downregulation of phospholipids in differentiated THP1 cells and A549 cells. Cells were infected with *S. aureus* strain SH1000 at an MOI of 25, harvested 2 h post infection, and lipids were then assayed using the same targeted lipidomic assays as for the human plasma samples. Values refer to mean fold concentration change with respect to uninfected cells. Bars = standard error of the mean (n = 3). **a** Phosphatidylcholines (PC). **b** Lysophosphatidylcholines (lysoPC). **c** Sphingomyelins (SM). **d** Acylcarnitines (AC). A549, adenocarcinoma cell line; THP1, myelomonocytic cell line; dTHP1, differentiated THP1 cells resembling human macrophages

Considering the well-documented property of CRP to bind to phospholipids [29], we investigated whether the downregulation observed above (Fig. 2a, b) was due to a technical artefact, i.e. the failure of our extraction procedure to capture phospholipids sequestered in complexes with CRP. In fact, adding increasing amounts of recombinant CRP to normal human plasma led to a trend towards increased concentrations, but clearly not to a decrease in any the four lipid classes (Additional file 1: Fig. S3). We then used *S. aureus* to infect human cell lines which represent important target cells in pneumonia (THP1 = monocytes; differentiated THP1 = macrophages; A549 = respiratory epithelial cells) and then determined infection-associated changes in lipid populations (Fig. 3). Of note, similar to the decrease in plasma concentrations, *S. aureus* infection led to a significant decrease in all three phospholipid classes in dTHP1 cells, and PC and SM in A549 cells. In contrast, no change was observed in AC, whose plasma concentrations were not significantly decreased. These data suggest that changes of phospholipids in host cells that are infected or exposed to the associated inflammation may contribute to the observed decrease in phospholipid plasma concentrations.

### Identification of diagnostic phospholipid biomarkers for CAP

**Fig. 4:**
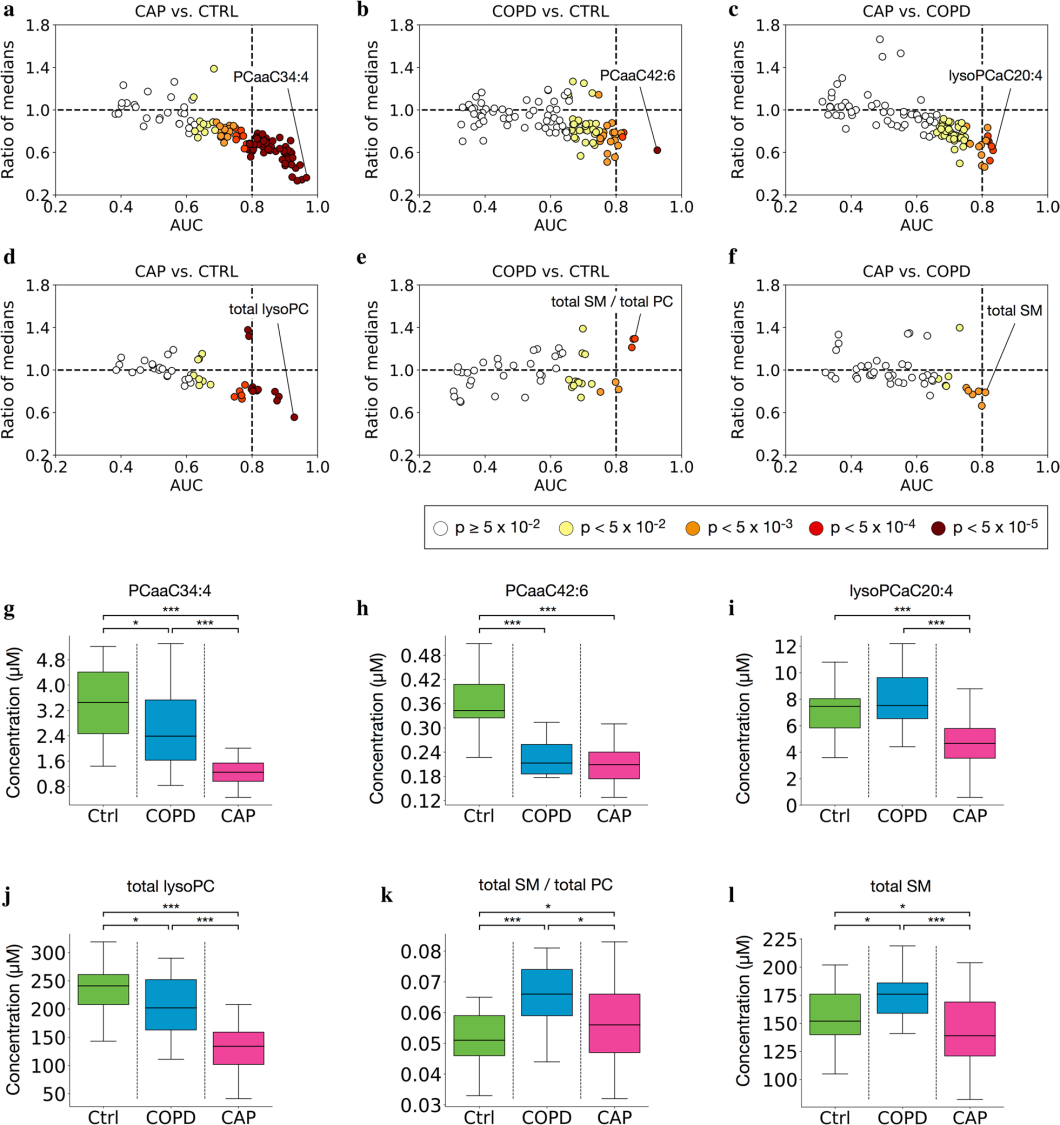
Identification of diagnostic biomarkers by ROC curve analysis. Differential expression (ratio of medians, also referred to as “fold change”; y-axis) is plotted against area under the ROC curve (AUC, x-axis), with darkness of fill color indicating the asymptotic significance of the ROC curve. Each dot corresponds to one lipid analyte (**a**–**c**) or one metabolite indicator (**d**–**f**). The dotted vertical line highlights the cut-off for “excellent classification” [26], i.e. AUC 0.80. **a**, **d** CAP baseline vs. Controls; **b**, **e** COPD vs. Controls; **c**, **f** CAP baseline vs. COPD The ROC analysis parameters of all markers with AUC ≥ 0.8 for each comparison are listed in Additional file 1: Table S2. **g**–**l** Levels of the most accurate biomarker for each of the three paired comparisons shown in **a**–**f**. **g**–**i** Best lipid biomarkers; PCaaC34:4 (CAP vs. CTRL), PCaaC42:6 (COPD vs. Control), lysoPCaC20:4 (CAP vs. COPD). **j**–**l** Best metabolic indicator biomarkers; Total lysoPC (CAP vs. Control), Total SM/Total PC (COPD vs Control), total SM (CAP vs. COPD). The box spans the 25th–75th percentile; lower whiskers = bottom quartile; upper whisker = upper quartile; and horizontal line = median. *p < 0.05, **p < 0.01, ***p < 0.001 (Mann–Whitney U test). Metabolite: lysoPC, lysophosphatidylcholines; PC, phosphatidylcholines; SM, sphingomyelins

Using binary ROC curve analysis, we then evaluated the biomarker potential of the lipid analytes and metabolic indicators at baseline for each of the three possible paired diagnostic comparisons (Fig. 4, Additional file 1: Table S3). Not unexpectedly, the highest number of accurate biomarkers according to the three criteria (AUC ≥ 0.8, lower 95% CI ≥ 0.5, and p < 0.05) was found for the discrimination between CAP (baseline) and Controls (n = 46 analytes, Fig. 4a), and a much lower number (n = 5) for the distinction COPD vs. Controls (Fig. 4b). Of note, four analytes fulfilling these criteria were identified even for the differentiation between the clinically relatively closely related diagnoses CAP and COPD (Fig. 4c). Consistent with the heat maps shown in Fig. 2a, there was a strong trend toward decreased concentrations in the more inflamed group (i.e. CAP > COPD and Controls; COPD > Controls) with respect to the less inflamed group; indeed, this was the case in all biomarkers fulfilling the three above criteria. As shown in detail in Additional file 1: Table S4, most of these biomarkers corresponded to PC (71%), followed by lysoPC (20%) and SM (9%), whereas there were no AC biomarkers. Assessing the biomarker potential within each class did reveal a higher potential of lysoPC in that a substantially higher percentage of lysoPC (10/13 = 77%) than PC (33/70 = 47%) constituted biomarkers according to the three criteria (Additional file 1: Table S4). Considering that much fewer biomarkers were identified for COPD, we tested whether this was due to the smaller sample size (13 COPD vs. 29 CAP). An over-sampling method in which the COPD sample size was modeled to be 29 (i.e. the same as of CAP) yielded 13 biomarkers (Additional file 1: Table S5), which was a consequence of improved prediction accuracy of the model resulting in tighter AUC confidence intervals and a higher number of features with AUC 95% CI lower bound > 0.5. Thus, the lower biomarker potential of COPD was predominantly due to disease-specific differences and only to a minor extent due to the smaller sample size. Considering the higher percentage of COPD patients undergoing systemic corticosteroid therapy, we also tested whether the differences between CAP and COPD could be results of corticosteroid treatment. However, multiple logistic regression analysis controlling for corticosteroid treatment did not reveal a significant effect (data not shown).

We also identified several metabolic indicators as accurate biomarkers for the differentiation between CAP vs. Controls (Fig. 4d), the five most accurate of which corresponded to the PC or lysoPC classes or ratios featuring them (Additional file 1: Table S3). Consistent with a previous report of sphingolipid induction in COPD [30], total SM showed some potential to distinguish between COPD and Controls, but significance was not achieved due to AUC CI crossing below 0.5 (Fig. 4e, Additional file 1: Table S3). However, due to the reciprocal regulation of total SM and some individual SM in COPD (up) and CAP (down) observed in Fig. 2, higher total SM accurately distinguished COPD from CAP (Fig. 4f, Additional file 1: Table S3).

For each of the three diagnostic groups, absolute concentrations of the most accurate single biomarker for each of the three comparisons are shown in Fig. 4g–i, and values of the most accurate metabolic indicator biomarker in Fig. 4j–l. Taken together, these results agreed with the PCA (Fig. 1) in that lipid concentrations changed most significantly in CAP but that there were substantial differences even between CAP and COPD.

### Normalization of lysoPC metabolism most closely parallels resolution of CAP

**Fig. 5:**
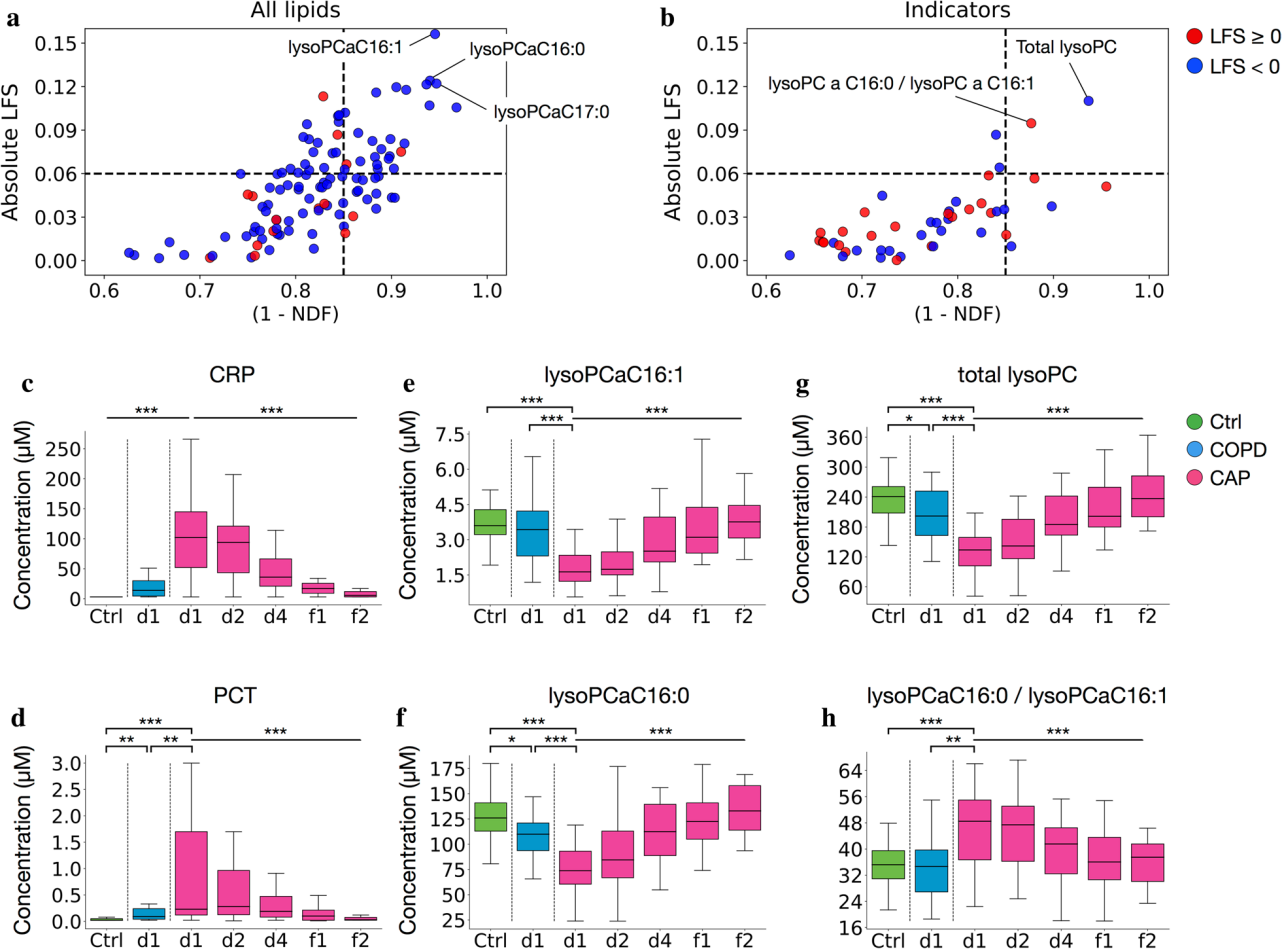
Selection of lipid biomarkers that correlate with resolution of CAP. A plot of linear fitting slope (y-axis, LFS) vs. 1-normalized distance to fitting (x-axis, NDF) was used to identify lipid analytes (**a**) and metabolic indicators (**b**) that correlate best with resolution of CAP. (C-H) Concentrations across the time course for CRP (**c**), PCT (**d**), the 2 best correlating lipids, lysoPCaC16:1 (**e**), lysoPCaC16:0 (**f**), and the 2 best correlating metabolic indicators, total lysoPC (**g**), lysoPCaC16:0/lysoPCaC16:1 (**h**). *p < 0.05, **p < 0.01, ***p < 0.001 (Mann–Whitney U test for paired comparisons, Kruskal–Wallis H-test for CAP d1 through CAP f2). Metabolite abbreviation: lysoPC, lysophosphatidylcholines

We then screened for changes in lipid concentrations and metabolic indicators in CAP throughout the time course that correlated best with resolution of inflammation and clinical improvement. In addition to clinical information (by which all patients with available blood samples had experienced resolution of symptoms by f1 and/or f2), the decline of CRP levels was used as a measure of inflammation resolution. This screen led to the identification of several phospholipids (Fig. 5a, Additional file 1: Table S6) and two metabolic indicators that correlated with resolution of CAP (Fig. 5b, Additional file 1: Table S6). Of these, 6 lysoPC had the steepest slope towards normalization, and the two metabolic indicators selected by this screen were based on lysoPC (Additional file 1: Table S6). Figure 5c–h illustrates the marked normalization of systemic inflammation (CRP, PCT = panels c, d), concentrations of the two best correlating phospholipid markers (lysoPCaC16:1 and lysoPCaC16:0 = panels e, f), and values of the two best correlating metabolic indicators (total lysoPC and lysoPCaC16:0/C16:1 = g, h) across the time course. Thus, normalization of lysoPC metabolism most closely paralleled resolution of inflammation and clinical remission in CAP.

### Increased ceramide synthesis and acid sphingomyelinase activity in CAP

**Fig. 6:**
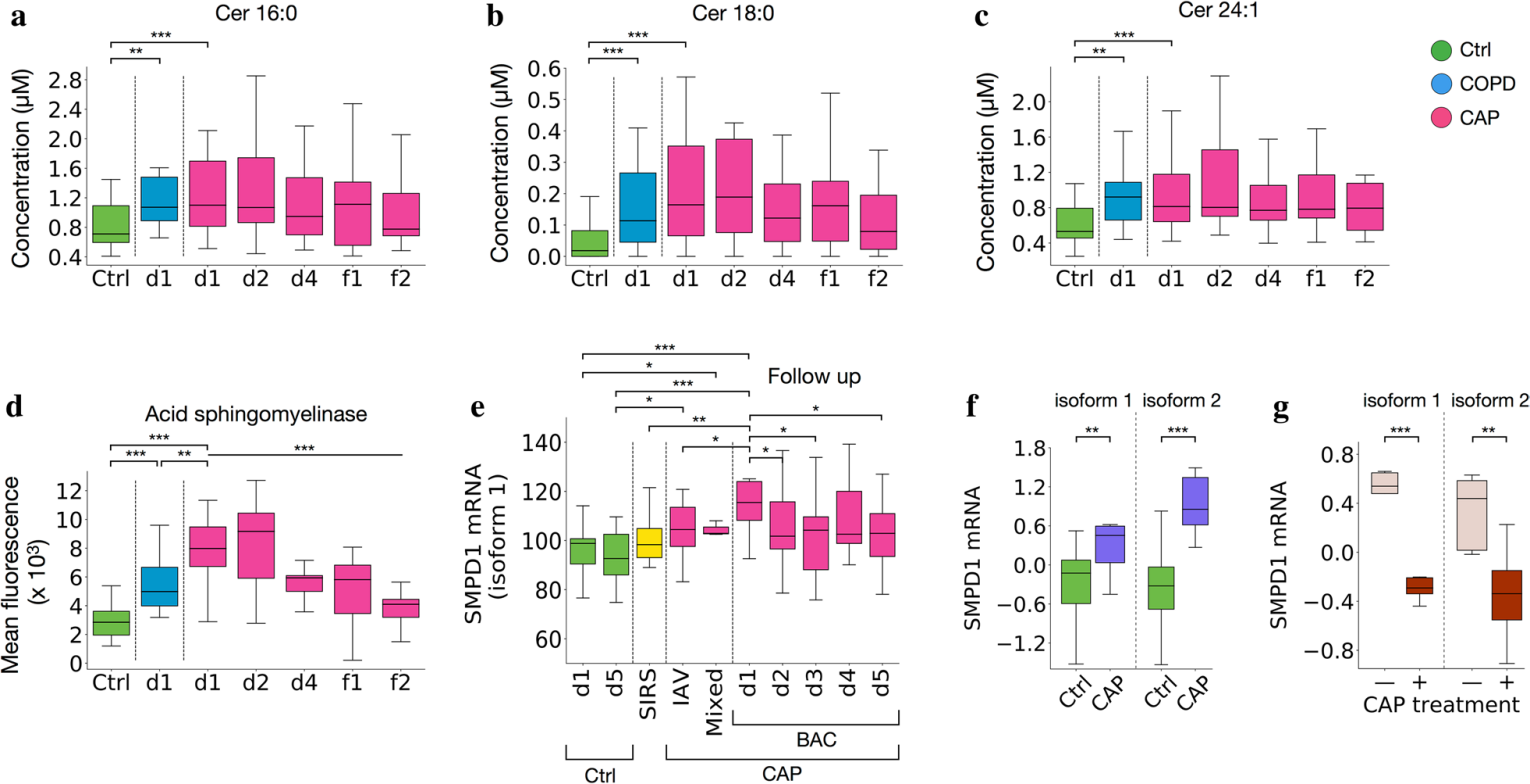
Ceramide synthesis and acid sphingomyelinase activity are increased in CAP and COPD exacerbation. **a** Ceramide C 16:0, **b** ceramide C 18:0, **c** ceramide C 24:1, **d** acid sphingomyelinase activity, measured with an in vitro assay based on separate aliquots from the plasma samples used for the mass-spectrometric measurements. **e**–**g** Increased expression of *SMPD1* (sphingomyelin phosphodiesterase, synonymous with acid sphingomyelinase) mRNA in CAP. Published datasets from studies on gene expression in CAP were reanalyzed for expression of *SMPD1* mRNA as described in [47]. The mRNA expression data had been obtained with microarray analysis of whole blood. Y-axis units correspond to normalized expression values. **e** Increased expression of splicing isoform 1 in community-acquired bacterial CAP [23], available at GEO, accession no GSE40012. Diagnostic groups: Bacterial CAP (BAC), n = 16; influenza A virus CAP (IAV), n = 8; mixed infection CAP (mixed, n = 3); systemic inflammatory response syndrome (SIRS, n = 12); healthy donors (HD, n = 18). **f**, **g** Increased expression of splicing isoforms 1 and 2 in CAP at baseline (**f**) and decrease of both isoforms after treatment of CAP (**g**) [24], GEO accession no. 42830. Diagnostic groups in **f**: Ctrl (healthy controls, n = 38), CAP (n = 8). Diagnostic groups in **g**: CAP before and after treatment, n = 5 each. *p < 0.05, **p < 0.01, ***p < 0.001 (Mann–Whitney U test for paired comparisons)

Even though they were not found among the accurate biomarkers (as defined in “Methods, materials and participants”), concentrations of some sphingomyelins (the precursors of ceramides) were decreased in CAP and, less so, in COPD. In addition, the close association of ceramide synthesis with inflammation has been well documented [12, 31]. We therefore used a dedicated mass spectrometry assay to measure concentrations of four ceramides which could potentially be derived from downregulated SM. Concentrations of ceramides C16:0, C18:0, and (less so) C24:1 were increased in CAP and normalized towards f2 (Fig. 6a–c), whereas no change was detected in C24:0 concentrations. Thus, even though there was a clear tendency toward increases in ceramides in CAP and COPD, distinct patterns were observed depending on the ceramide in question. Considering the potential conversion of SM to ceramides in CAP and, less so, COPD, we then tested whether activity of the corresponding enzyme, acid sphingomyelinase, was increased. Indeed, enzyme activity was 2.8-fold (median) higher in CAP than in Controls and normalized towards f2, whereas the increase in COPD was less pronounced (1.75-fold, median) (Fig. 6d). Of note, enzyme activity was an accurate biomarker for both CAP at baseline (AUC, 0.94 [0.81–1.0]) and COPD (AUC, 0.88 [0.62–1.0]) with respect to Controls. Since there are no other reports of acid sphingomyelinase activity in CAP to corroborate our finding, we interrogated published data sets of whole blood mRNA expression in different forms of CAP and control groups for expression of the acid sphingomyelinase gene (sphingomyelin phosphodiesterase 1, *SMPD1*, OMIM: 607608). Indeed, expression of *SMPD1* splicing isoform 1 was significantly higher in bacterial CAP compared to controls and began to normalize during 5 days of treatment (Fig. 6e), but expression of isoform 2 was not significantly altered in bacterial CAP (data not shown) [23]. In contrast, in a different cross-sectional study, expression of both isoforms was elevated (Fig. 6f) [24]. Of note, in the same study expression of both isoforms also decreased significantly after treatment of CAP (Fig. 6g). Thus, disease-relevant regulation of acid sphingomyelinase expression in CAP was clearly evident also at the mRNA level.

### Modest correlations with markers of systemic inflammation

**Fig. 7:**
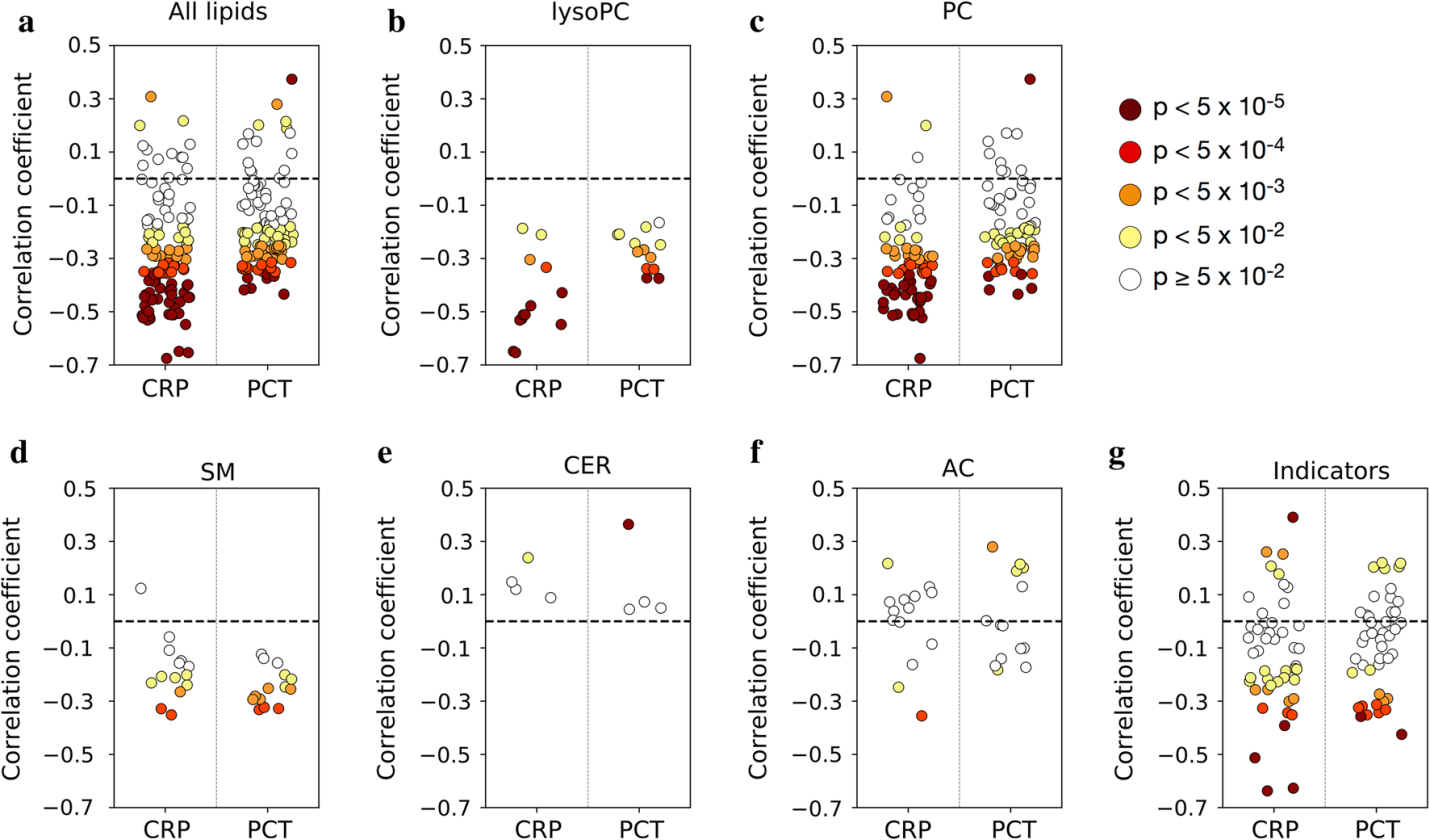
Correlation analysis of lipid analytes or metabolic indicators for CAP d1 through f2 with CRP and PCT. Y-axis values correspond to Pearson’s rho; significance of p values (Mann–Whitney U test) is indicated by the fill color of the circles. **a** 111 lipid analytes measured with the MetIDQ p180 kits. **b** lysopc; **c** PC; **d** SM; **e** ceramides (measured with targeted assays); **f** AC; and **g** metabolic indicators. Metabolite: AC, acylcaritines; Cer, ceramides; lysoPC, lysophosphatidylcholines; PC, phosphatidylcholines; SM, sphingomyelins

We then performed a correlation analysis in CAP in order to test to what extent the lipids and metabolic indicators merely reveal processes already captured by CRP. Across all lipids there was a striking negative correlation of the majority with CRP (79 of 111 [71%] lipids at p < 0.05) and PCT (69 of 111, 62%), with negative correlations with PCT tending to be weaker (Fig. 7a, Additional file 1: Table S7). In particular, correlations of lysoPC and SM were exclusively negative (Fig. 7b, d), whereas the scattered positive correlations stemmed from PC (Fig. 7c), ceramides (Fig. 7e, Additional file 1: Table S7), and AC (Fig. 7f) and were of much smaller magnitude. Acid sphingomyelinase activity correlated moderately with both CRP (r = 0.29, p = 9.36E−04) and PCT concentrations (r = 0.29, p = 1.04E−03). Of note, the best biomarker for CAP identified with the ROC analysis (PCaaC34:4, Fig. 4a) correlated only moderately (r = − 0.51) with CRP (Additional file 1: Table S7). Correlations of the metabolic indicators also tended to be negative (Fig. 7g, Additional file 1: Table S7), but compared to the lipids the proportion of significant negative correlations were smaller for both CRP (21 of 44 [48%] correlations had p < 0.05) and PCT (13 of 44, 30%), as compared to the individual analytes. Thus, in spite of an obvious association with systemic inflammation (which is also, for instance, indicated in Fig. 5), the marked changes in lipid metabolism clearly also reflect other aspects of pathogenesis.

## Discussion

We have performed a targeted metabolomic analysis of 111 plasma lipids in CAP, which distinguishes itself from other lipidomic studies of CAP [19, 20] in that (1) comparisons were made against COPD exacerbation with infection as a clinically highly relevant disease control, (2) serial measurements were obtained in CAP allowing to assess changes in lipid concentrations during clinical recovery and resolution of inflammation, and (3) salient results were verified in a cellular infection model.

### Marked decrease in plasma phospholipid concentrations

We observed a striking decrease of phospholipid concentrations in acute CAP, which by and large normalized with clinical recovery. The correlation analysis clearly showed that this is not merely a linear reflection of systemic inflammation. There are different, not mutually exclusive, explanations for this downregulation. Firstly, it has been suggested that phospholipids can bind to acute phase reactants including CRP [29], but we have ruled out that this may lead to an artefactual decrease in our assay (Additional file 1: Fig. S3). Thus, subsequent removal of the complex (e.g. in the reticuloendothelial system) would be required in order to lead to reduced concentrations measured with our assay. Secondly, the balance between synthesis, incorporation into cell building blocks, release from cell turnover, and catabolism to downstream products may change greatly in acute infection. PCs can be hydrolyzed by phospholipase A2 releasing one fatty acid and a lysoPC, which can then be hydrolyzed by lysophospholipase to yield another free fatty acid. Another explanation could be the oxidation of phospholipid side chains. During oxidative stress and inflammation, polyunsaturated fatty acid side chains of membrane phospholipids can be modified by oxidation [32]. The best biomarkers for both CAP and COPD (Additional file 1: Table S3, Fig. 4) contain poly-unsaturated fatty acids; for instance, PCaaC34:4 could contain arachidonic acid (C20:4), which can be oxidized to prostaglandins, i.e. well known players in the inflammatory response [33]. Indeed, the PCaaC34:4 that contains arachidonic acid in addition to myristic acid (C14) makes of 37% of PCaaC34:4 species in human plasma [34]. Oxidized phospholipids can also bind to the scavenger receptor CD36 (a fatty acid translocase), be internalized into cells, and then serve as energy substrate for beta-oxidation, for instance in T cells [35].

Decreased blood phospholipid concentrations have been documented in other invasive bacterial infections (bacteremia [36], sepsis [37]), and also in CAP [19, 20]. In particular, the recent study by Müller et al. [20] found markedly decreased lysoPC concentrations in CAP on admission, which normalized towards convalescence. These authors also demonstrated that the decreased lysoPC concentrations were neither due to metabolism by autotaxin nor reduced dietary intake during the acute illness. Furthermore, based on previous publications they suggested that the depressed lysoPC concentrations may adversely affect host defenses in CAP and that pharmacologically raising them might be a host-directed treatment for CAP. In the present work, we show that the decreased phospholipid concentrations are not due to a technical artefact resulting from mere binding to CRP, but that phospholipid concentrations decrease in *S. aureus* infected human cells. This effect was most pronounced in dTHP1 cells, which resemble activated macrophages found in human tissues including the lung. Importantly, *S. aureus* infection did not affect AC concentrations, demonstrating that the infection did not exert a global effect on cellular lipids. Taken together, our data suggest that at least part of the observed decreased concentrations in plasma may be preprogrammed at the level of infected cells, for instance due to activity of phospholipases. We did not measure activity of enzymes other than acid sphingomyelinase, but it is conceivable that the final phospholipid concentrations result from an intricate network of intracellular and secreted enzymes. Our results partially differ from the bacteremia study by To et al., who used untargeted metabolomics and found elevated concentrations of lysoPC 18:1 (which was clearly downregulated in our study) and SM 18:0/16:0 (which were upregulated in COPD, but essentially unchanged in our study) in CAP [36]. These discrepancies may be due to differences in the detection assays, preanalytical sample processing, or in the clinical population. Clearly, much additional work is still needed to unravel the causes and consequences of depressed phospholipid concentrations in CAP and other acute infections.

### Implications of increased acid sphingomyelinase activity

Acid sphingomyelinase activity was markedly increased in CAP at baseline and decreased steadily throughout the time course, suggesting that the marked increase at presentation is clinically relevant. Sphingomyelins are ubiquitous cellular components and are involved in a variety of processes including cell division, cell proliferation and autophagy [38] and help to establish an equilibrium between pro- and anti-inflammatory lipids and also to regulate immune responses in lung tissues [39], whereas ceramides are predominantly involved in inflammation- and damage-associated processes [40]. Concentrations of 5/13 (38%) of the SM were not decreased, but the sum of SM was significantly lower in CAP, suggesting a broad effect upon SM concentrations. On the other hand, concentrations of only two of the four measured ceramides increased substantially in CAP. Considering that we profiled only a fraction of cellular SM and ceramides, and that there are several other enzymes that mediate ceramide synthesis [41], it is possible that the induction of ceramide synthesis is much more extensive than can be gleaned from our results. Pharmacological inhibition of acid sphingomyelinase in a mouse model of *S. aureus* sepsis reduced liver damage, pulmonary edema, and mortality [12, 13], inhaled sphingomyelinase inhibitors reduced airway inflammation and *Pseudomonas aeruginasa* infection in a mouse model of cystic fibrosis [14], and this enzyme has thus been proposed as a target of host-directed treatments for bacterial infections and other inflammation-associated diseases [16, 17]. Our study now provides evidence of a clinically relevant increase in its activity in CAP and, thus, suggests acid sphingomyelinase as a potential target for host directed treatments to reduce end-organ damage in pneumonia. The increase in enzyme activity in COPD exacerbation was not as pronounced, but it is possible that acid sphingomyelinase inhibition might also be promising for a subgroup of COPD patients with the highest enzyme active, possibly manifested as unusually high ceramide levels.

### Identification of diagnostic and correlative biomarkers for CAP

Our analysis revealed several highly accurate plasma lipid biomarkers for acute CAP as compared with Controls. The top 5 corresponded to PCs, which agreed well with the marked decrease observed in this class. It was not possible to compare their diagnostic accuracy to that of CRP because elevated CRP had been used as part of the case definition of CAP. However, the results do suggest that plasma phospholipids have strong biomarker potential for CAP, and future studies should be geared toward evaluating their ability to differentiate between different subtypes of CAP, e.g. by bacterial pathogen, bacterial vs. viral etiologies, or different risk classes, or to aid in decision-making regarding stopping or escalating antibiotic treatment. Of note, several biomarkers (although somewhat less accurate) were also identified for the clinically important differentiation between CAP and infection-associated COPD exacerbation, and there was only partial overlap with the top biomarkers for CAP vs. Controls. In addition, yet different PC species constituted the best biomarkers for COPD vs. Controls. These results suggest (1) that plasma phospholipids merit further investigation as biomarkers to help differentiate between CAP and COPD (or to potentially identify COPD patients with coexisting pneumonia) and (2) that the observed differences between CAP and COPD are not merely due to differences in the extent of inflammation-driven lipid reprogramming in the same direction, but that they also reflect inherent differences in pathophysiology between the two disease entities. Indeed, the heat maps shown in Fig. 2 also illustrate clear qualitative differences in the lipid concentration patterns of COPD and CAP as compared with Controls. For instance, total SM and some individual SM were upregulated in COPD exacerbation only, which agrees well with previous reports of increased SM in COPD [30].

Curiously, regulation of AC concentrations differed from the other lipid classes in that the overall degree of change was the lowest and that only one (the short-chain AC valerylcarnitine, C5) fulfilled criteria for differential abundance, but did not correlate with CRP or PCT levels. Studies in sepsis have suggested that increased levels of peripheral blood short-chain AC reflect organ damage, notably of liver and kidney, and that increased acetyl carnitine (C2) concentrations are associated with mortality [18]. Animal models of sepsis have suggested that this may be due to an acquired defect in fatty acid oxidase [42]. Considering that none of our patients had poor outcomes, it remains to be studied whether ACs can be used to assess organ damage or clinical outcome in more severe CAP.

### Potential impact of diet, age and sex

In agreement with the above mentioned studies on lipid biomarkers in acute infections, we did not collect data on food intake. Clinical experience shows that food intake is often markedly reduced in patients with acute illness, due to factors such as loss of appetite, physical weakness, or a medical indication for fasting status. It is therefore conceivable that reduced food intake in the CAP and COPD groups (compared to the control subjects, who were healthy or presented to hospital for elective procedures) might have introduced a bias. However, we detected clear differences in lipid profiles even between these two acutely ill groups. In addition, it has been argued (perhaps contrary to popular belief) that effects of fasting status on blood metabolite profiles are relatively weak and explain only a small fraction of metabolite variability [43]. Indeed, in a large study (n = 1197) no effects of fasting on saturated, mono- or poly-unsaturated phosphatidylcholines were detected [44], but total poly-unsaturated phosphatidylcholines were clearly reduced in acute CAP in our study (Fig. 2c). In the same report, AC were found to be affected by fasting status, but in our study showed the least variability among the three groups. In addition, in the aforementioned study on phospholipids in CAP, Müller et al. found no differences in the concentrations of the assessed 75 phospholipids after a 14-h fast [20]. Taken together, these results indicate that, while we cannot quantify the effects of differences in food intake on our results, they are likely to be minor compared to those of the primary diseases, CAP and COPD exacerbation. Age and sex are well documented variables affecting concentrations of several parameters measured with the Biocrates p180 kits [27], but both were well matched among the three groups in our study.

### Limitations and strengths

Even though our results agree well with published evidence, this study is clearly limited by the lack of an external validation cohort and the relatively small sample sizes. Further research is therefore needed before the identified biomarkers can be advanced to clinical application. This is particularly true for the COPD exacerbation group, so that the results regarding COPD should be interpreted cautiously, including the diagnostic biomarker potential of the assayed lipid classes to differentiate infection-associated COPD exacerbation from CAP or to detect pneumonia in COPD exacerbation. In addition, the study was not designed to assess longer-term outcomes such as mortality after resolution of CAP, and due to the small proportion of patients with severe disease it was not feasible to screen for prognostic biomarkers for complications. Considering that the human plasma lipidome contains at least 600 named and quantifiable lipid species [45] (but probably many more [46]), our assays captured only a small part of the possible spectrum. On the other hand, our study differs from the other studies on phospholipids in CAP [19, 20] by the analysis of a broader spectrum of lipid classes and species, use of a well-matched control group, inclusion of infection-associated COPD exacerbation as a clinically important disease control, and serial measurements within a relatively narrow window after disease presentation. Moreover, to our knowledge it provides the first evidence of increased acid sphingomyelinase activity in pneumonia and COPD exacerbation in humans.

## Conclusions

Our results clearly suggest that plasma phospholipids merit further evaluation as biomarkers for CAP as well as COPD, for instance by external validation of specific biomarkers in larger, independent cohorts that are designed to address subgroup-specific outcomes. Moreover, it will now be of great interest to investigate how the balance between different phospholipid populations affects disease severity and organ damage and whether therapeutic interventions, such as transiently inhibiting acid sphingomyelinase activity to reduce levels of potentially damaging ceramides, will improve clinical outcome.

## Supplementary information

**Additional file 1: Figure S1.** Efficiency of analyte detection across all samples. The percentage of samples in which the analytes were detected > LOD is indicated by the fill darkness. All analytes detected > LOD in ≥ 75% of the samples (black and dark grey fill) were included in subsequent analyses. **(A)** Of the 145 potentially detectable lipids, 111 were detected > LOD in ≥ 75% of the samples. **(B**) The same lipids as in (A) but separated by lipid class. Detection efficiency was greatest for glycerophospholipids and sphingolipids. **Figure S2.** Contribution of each lipid analyte and indicator to the principal components PC1 and PC2, and the square root of the sum of their squares. The higher the absolute value, the higher the influence on the principal component. (A) Analysis based on concentrations of all 111 lipid analytes; (B) PC only; (C) SM only; (D) AC only; (E) lysoPC only; and (F) 47 metabolic indicators. Results are arranged in descending order according to the PC’s values. **Figure S3.** Exogenously increasing CRP concentrations does not decrease measured plasma lipid concentrations. Normal human plasma (n = 3 replicates per treatment) was incubated with increasing concentrations of recombinant CRP, extracted with 85% EtOH/15% PBS and then analyzed for concentrations of 145 lipids using the Biocrates AbsoluteIDQ^®^ p180 kit. Increasing CRP concentrations are not associated with decreased concentrations of any of the four lipid classes. **(A)** phosphatidylcholines, **(B)** lysophosphatidylcholines, **(C)** sphingomyelins, and **(D)** acylcarnitines and tables.

## Data Availability

The data used and/or analyzed are available from the corresponding author on a reasonable request.

## Abbreviations

AC: acylcarnitine
AUC: area under the curve
CAP: community-acquired pneumonia
COPD: chronic obstructive pulmonary disease
CRP: C-reactive protein
FDR: false discovery rate
LOD: limit of detection
lysoPC: lysophosphatidylcholine
PC: phosphatidylcholine
PCA: principal component analysis
PCT: procalcitonin
PSI: Pneumonia Severity Index
ROC: receiver operating characteristic
SM: sphingomyelin
