# Supplementary material for: Decreased plasma phospholipid concentrations and increased acid sphingomyelinase activity are accurate biomarkers for community-acquired pneumonia
Source: J Transl Med. 2019 Nov 11;17:365. doi: 10.1186/s12967-019-2112-z (PMC6849224; doi:10.1186/s12967-019-2112-z)
Supplement: Supplementary file 1 — Additional file 1: Figure S1. Efficiency of analyte detection across all samples. The percentage of samples in which the analytes were detected > LOD is indicated by the fill darkness. All analytes detected > LOD in ≥ 75% of the samples (black and dark grey fill) were included in subsequent analyses. (A) Of the 145 potentially detectable lipids, 111 were detected > LOD in ≥ 75% of the samples. (B) The same lipids as in (A) but separated by lipid class. Detection efficiency was greatest for glycerophospholipids and sphingolipids. Figure S2. Contribution of each lipid analyte and indicator to the principal components PC1 and PC2, and the square root of the sum of their squares. The higher the absolute value, the higher the influence on the principal component. (A) Analysis based on concentrations of all 111 lipid analytes; (B) PC only; (C) SM only; (D) AC only; (E) lysoPC only; and (F) 47 metabolic indicators. Results are arranged in descending order according to the PC’s values. Figure S3. Exogenously increasing CRP concentrations does not decrease measured plasma lipid concentrations. Normal human plasma (n = 3 replicates per treatment) was incubated with increasing concentrations of recombinant CRP, extracted with 85% EtOH/15% PBS and then analyzed for concentrations of 145 lipids using the Biocrates AbsoluteIDQ® p180 kit. Increasing CRP concentrations are not associated with decreased concentrations of any of the four lipid classes. (A) phosphatidylcholines, (B) lysophosphatidylcholines, (C) sphingomyelins, and (D) acylcarnitines and tables. [file 12967_2019_2112_MOESM1_ESM.docx]

**Additional Figures**

**Figure S1: Efficiency of analyte detection across all samples.** The percentage of samples in which the analytes were detected >LOD is indicated by the fill darkness. All analytes detected >LOD in ≥75% of the samples (black and dark grey fill) were included in subsequent analyses. (A) Of the 145 potentially detectable lipids, 111 were detected >LOD in ≥75% of the samples. (B) The same lipids as in A but separated by lipid class. Detection efficiency was greatest for glycerophospholipids and sphingolipids.

**Figure S2 (to Fig. 1, PCA): Contribution of each lipid analyte and indicator to the principal components PC1 and PC2, and the square root of the sum of their squares (PC’s)**. The higher the absolute value, the higher the influence on the principal component. (A) Analysis based on concentrations of all 111 lipid analytes; (B) PC only; (C) SM only; (D) AC only; (E) lysoPC only; and (F) 47 metabolic indicators. Results are arranged in descending order according to the PC’s values.

**Figure S3: Exogenously increasing CRP concentrations do not decrease measured plasma lipid concentrations.** Normal human plasma (n=3 replicates per treatment) was incubated with increasing concentrations of recombinant CRP, extracted with 85% EtOH/15% PBS and then analyzed for concentrations of 145 lipids using the Biocrates AbsoluteIDQ® p180 kit. Increasing CRP concentrations are not associated with decreased concentrations of any of the four lipid classes. (A) phosphatidylcholines, (B) lysophosphatidylcholines, (C) sphingomyelins, and (D) acylcarnitines.

**Additional Tables**

| **Table S1. List of metabolic indicators*** | | **Description** | **Metabolic significance**** |
| --- | --- | --- | --- |
| **Sums (mean concentrations)** | |  |  |
| 1 | Total lysoPC | Sum of lysophosphatidylcholines | n/s |
| 2 | Total PC ae | Sum of phosphatidylcholine plasmalogens | n/s |
| 3 | Total SM-OH | Sum of hydroxylated sphingomyelins | n/s |
| 4 | PUFA (PC) | Sum of poly-unsaturated phosphatidylcholines | Indicator of nutritional lipid composition |
| 5 | Total SM | Sum of sphingomyelins | n/s |
| 6 | Total SM-non OH | Sum of non-hydroxylated sphingomyelins | n/s |
| 7 | Total (PC+SM) | Sum of choline-containing phospholipids | n/s |
| 8 | Total PC | Sum of phosphatidylcholines | n/s |
| 9 | MUFA (PC) | Mono-unsaturated phosphatidylcholines | Indicator of nutritional lipid composition, lipid peroxidation, cell signalling |
| 10 | SFA (PC) | Sum of saturated phosphatidylcholines | Indicator of nutritional lipid composition, cell signalling |
| 11 | Total PC aa | Sum of diacyl-phosphatidylcholines | n/s |
|  | |  |  |
|  | **Ratios involving sums** |  |  |
| 1 | CPT-I ratio | Ratio of long-chain acylcarnitines to free carnitine ([C16+C18]/C0) | Activity of carnitine palmytoyl transferase I, rate-limiting step in the uptake of fatty acids into mitochondria |
| 2 | MUFA (PC) / SFA (PC) | Ratio of mono-unsaturated to saturated phosphatidylcholines | Measure of the activity of fatty acid desaturases and indicator of nutritional lipid composition, lipid peroxidation, cell signalling |
| 3 | PUFA (PC) / MUFA (PC) | Ratio of poly-unsaturated to mono-unsaturated to saturated phosphatidylcholines | Measure of the activity of fatty acid desaturases and indicator of nutritional lipid composition, lipid peroxidation, cell signalling |
| 4 | PUFA (PC) / SFA (PC) | Ratio of poly-unsaturated to saturated phosphatidylcholines | Measure of the activity of fatty acid desaturases and indicator of nutritional lipid composition, lipid peroxidation, cell signalling |
| 5 | Total AC / C0 | Ratio of esterified to free carnitine | n/s |
| 6 | Total AC-DC / Total AC | Fraction of dicarboxylacylcarnitines of total acylcarnitines | Indicator of ω-oxidation of fatty acids |
| 7 | Total AC-OH / Total AC | Fraction of hydroxylated acylcarnitines of total acylcarnitines | n/s |
| 8 | Total lysoPC / Total PC | Ratio of lysophosphatidylcholines to phosphatidylcholines | Phospholipase activity |
| 9 | Total SM / Total PC | Ratio of total sphingomyelins to total phosphatidylcholines | n/s |
| 10 | Total SM / Total (SM+PC) | Fraction of sphingomyelins of total phospholipid | n/s |
| 11 | Total SM-OH / Total SM-non OH | Ratio of hydroxylated to non-hydroxylated sphingomyelins | n/s |
|  | |  |  |
|  | **Ratios of individual lipid analytes** |  |  |
| 1 | C2 / C0 | Ratio of acetylcarnitine to free carnitine | Measure of β-oxidation of even numbered fatty acids |
| 2 | (C2+C3) / C0 | Ratio of short-chain acetylcarnitines to free carnitine | Measure of overall β-oxidation activity |
| 3 | C3 / C4 | Ratio of propionylcarnitine to butyryl-L-carnitine | n/s |
| 4 | C4 / C0 | Ratio of butyrylcarnitine to free carnitine | n/s |
| 5 | C4 / C5 | Ratio of butyryl-L-carnitine to valeryl-L-carnitine | n/s |
| 6 | C9 / C14 | Ratio of nonayl-L-carnitine to tetradecanoyl-L-carnitine | n/s |
| 7 | C9 / C10:2 | Ratio of nonayl-L-carnitine to decadienyl-L-carnitine | n/s |
| 8 | C9 / C14:1-OH | Ratio of nonayl-L-carnitine to hydroxytetradecenoyl-L-carnitine | n/s |
| 9 | C9 / C16:1-OH | Ratio of nonayl-L-carnitine to hydroxyhexadecenoyl-L-carnitine | n/s |
| 10 | C12 / C8 | Ratio of dodecanoyl-L-carnitine to octanoyl-L-carnitine | n/s |
| 11 | C12 / C10 | Ratio of dodecanoyl-L-carnitine to decanoyl-L-carnitine | n/s |
| 12 | C12 / C12:1 | Ratio of dodecanoyl-L-carnitine to dodecenoyl-L-carnitine | n/s |
| 13 | C14 / C16:1 | Ratio of tetradecanoyl-L-carnitine to hexadecenoyl-L-carnitine | n/s |
| 14 | C14:1-OH / C10 | Ratio of tetradecenoyl-L-carnitine to decanoyl-L-carnitine | n/s |
| 15 | C16 / C16:1 | Ratio of hexadecanoyl-L-carnitine to hexadecenoyl-L-carnitine | n/s |
| 16 | C18 / C18:1 | Ratio of octadecanoyl-L-carnitine to octadecenoyl-L-carnitine | n/s |
| 17 | lysoPCaC16:0 / lysoPCaC16:1 | Ratio of lysophosphatidylcholine acyl C16:0 to lysophosphatidylcholine acyl C16:1 | n/s |
| 18 | lysoPCaC20:4 / lysoPCaC20:3 | Ratio of lysophosphatidylcholine acyl C20:4 to lysophosphatidylcholine acyl C20:3 | n/s |
| 19 | PCaaC28:1 / PcaaC38:1 | Ratio of phosphatidylcholine diacyl C 28:1 to phosphatidylcholine diacyl C 38:1 | n/s |
| 20 | PcaaC28:1 / PcaaC40:2 | Ratio of phosphatidylcholine diacyl C 28:1 to phosphatidylcholine diacyl C 40:2 | n/s |
| 21 | PcaaC36:3 / PcaaC36:4 | Ratio of phosphatidylcholine diacyl C 36:3 to phosphatidylcholine diacyl C 36:4 | n/s |
| 22 | PcaaC40:3 / PcaaC42:5 | Ratio of phosphatidylcholine diacyl C 40:3 to phosphatidylcholine diacyl C 42:5 | n/s |
| 23 | PcaaC40:5 / PcaaC42:5 | Ratio of phosphatidylcholine diacyl C 40:5 to phosphatidylcholine diacyl C 42:5 | n/s |
| 24 | PcaeC32:1 / PcaeC34:1 | Ratio of phosphatidylcholine acyl-alkyl C 32:1 to phosphatidylcholine acyl-alkyl C 34:1 | n/s |
| 25 | PcaeC44:5 / PcaeC42:5 | Ratio of phosphatidylcholine acyl-alkyl C 44:5 to phosphatidylcholine acyl-alkyl C 42:5 | n/s |
| * Adapted from ref. 21; completed to include 23 additional ratios not considered in that reference.  **If indicative of a specific metabolic process or alteration, according to ref. 21 and additional literature search. | | | |

| **Table S2. Completeness of CAP study visits and blood samples** | | | | | |
| --- | --- | --- | --- | --- | --- |
| **Patient** | **d1** | **d2** | **d4** | **f1** | **f2** |
| 1 | X | X | — | X | X |
| 2 | X | X | — | X | X |
| 3 | X | X | X | — | — |
| 4 | X | X | X | X | X |
| 5 | X | X | X | X | X |
| 6 | X | X | X | X | X |
| 7 | X | X | X | X | X |
| 8 | X | X | X | X | X |
| 9 | X | X | X | X | X |
| 10 | X | X | X | X | — |
| 11 | X | X | — | X | — |
| 12 | X | X | X | X | — |
| 13 | X | X | X | X | — |
| 14 | X | X | X | X | X |
| 15 | X | X | X | X | — |
| 16 | X | — | — | — | X |
| 17 | X | X | X | — | X |
| 18 | X | X | X | X | X |
| 19 | X | X | X | X | X |
| 20 | X | X | X | X | — |
| 21 | X | X | X | X | X |
| 22 | X | X | X | X | X |
| 23 | X | X | X | X | X |
| 24 | X | X | X | X | — |
| 25 | X | — | X | — | X |
| 26 | X | X | — | — | X |
| 27 | X | X | X | X | — |
| 28 | X | X | X | X | — |
| 29 | X | X | X | X | X |
| **Percent** | 100 | 93 | 83 | 83 | 66 |

| **Table S3 (to Fig. 4A-F): Identification of diagnostic lipid biomarkers by ROC curve analysis.** The symbol * in the Lipid/Indicator column indicates accurate biomarkers as defined by AUC ≥ 0.8, asymptotic p < 0.05 and AUC lower bound 95% CI ≥ 0.5. | | | | |
| --- | --- | --- | --- | --- |
| **Fig. 4A (CAP vs. Ctrl)** | | | | |
| **Rank** | **Lipid** | **Mean AUC [95% CI]** | **Ratio of medians** | **p-value** |
| 1 | PCaaC34:4* | 0.97 [0.88, 1.0] | 0.36 | 1.36E-10 |
| 2 | PCaaC32:2* | 0.95 [0.83, 1.0] | 0.34 | 5.99E-10 |
| 3 | PCaeC40:1* | 0.95 [0.83, 1.0] | 0.48 | 7.44E-10 |
| 4 | PCaaC36:6* | 0.94 [0.80, 1.0] | 0.33 | 1.70E-09 |
| 5 | lysoPCaC16:1* | 0.93 [0.77, 1.0] | 0.45 | 3.34E-09 |
| 6 | PCaeC36:3* | 0.92 [0.76, 1.0] | 0.55 | 5.28E-09 |
| 7 | lysoPCaC18:2* | 0.92 [0.70, 1.0] | 0.37 | 4.68E-09 |
| 8 | PCaaC34:3* | 0.92 [0.78, 1.0] | 0.59 | 5.08E-09 |
| 9 | PCaeC38:0* | 0.92 [0.78, 1.0] | 0.48 | 7.68E-09 |
| 10 | PCaeC38:2* | 0.92 [0.74, 1.0] | 0.71 | 9.79E-09 |
| 11 | lysoPCaC18:0* | 0.92 [0.73, 1.0] | 0.5 | 8.68E-09 |
| 12 | PCaaC42:6* | 0.92 [0.76, 1.0] | 0.61 | 8.33E-09 |
| 13 | lysoPCaC16:0* | 0.91 [0.72, 1.0] | 0.59 | 1.02E-08 |
| 14 | PCaeC34:2* | 0.91 [0.73, 1.0] | 0.62 | 2.03E-08 |
| 15 | lysoPCaC17:0* | 0.91 [0.72, 1.0] | 0.55 | 1.87E-08 |
| 16 | lysoPCaC18:1* | 0.91 [0.67, 1.0] | 0.53 | 2.03E-08 |
| 17 | PCaaC36:3* | 0.90 [0.71, 1.0] | 0.65 | 3.01E-08 |
| 18 | PCaeC34:3* | 0.90 [0.72, 1.0] | 0.5 | 3.67E-08 |
| 19 | lysoPCaC20:3* | 0.90 [0.67, 1.0] | 0.48 | 3.14E-08 |
| 20 | PCaaC32:3* | 0.90 [0.71, 1.0] | 0.6 | 4.65E-08 |
| 21 | PCaeC36:5* | 0.90 [0.74, 1.0] | 0.61 | 5.01E-08 |
| 22 | PCaaC36:0* | 0.89 [0.64, 1.0] | 0.53 | 6.10E-08 |
| 23 | PCaeC38:6* | 0.88 [0.67, 1.0] | 0.64 | 1.40E-07 |
| 24 | PCaaC42:4* | 0.88 [0.70, 1.0] | 0.74 | 1.30E-07 |
| 25 | lysoPCaC28:1* | 0.87 [0.67, 1.0] | 0.61 | 2.95E-07 |
| 26 | PCaaC40:3* | 0.86 [0.67, 1.0] | 0.71 | 3.81E-07 |
| 27 | PCaaC40:2* | 0.86 [0.64, 1.0] | 0.65 | 6.75E-07 |
| 28 | SM (OH) C22:1* | 0.85 [0.67, 1.0] | 0.62 | 1.10E-06 |
| 29 | lysoPCaC24:0* | 0.85 [0.62, 1.0] | 0.63 | 9.23E-07 |
| 30 | SM C24:0* | 0.85 [0.62, 1.0] | 0.63 | 1.10E-06 |
| 31 | PCaaC28:1* | 0.85 [0.64, 1.0] | 0.65 | 1.91E-06 |
| 32 | PCaaC36:2* | 0.84 [0.57, 1.0] | 0.73 | 1.79E-06 |
| 33 | PCaaC42:2* | 0.84 [0.58, 0.98] | 0.69 | 1.40E-06 |
| 34 | PCaeC32:2* | 0.84 [0.57, 1.0] | 0.6 | 3.16E-06 |
| 35 | PCaeC36:2* | 0.84 [0.62, 1.0] | 0.72 | 3.17E-06 |
| 36 | PCaeC42:3* | 0.84 [0.60, 1.0] | 0.71 | 2.68E-06 |
| 37 | PCaaC38:5* | 0.83 [0.61, 1.0] | 0.68 | 2.59E-06 |
| 38 | PCaaC38:3* | 0.83 [0.64, 0.98] | 0.66 | 4.00E-06 |
| 39 | PCaeC38:5* | 0.83 [0.62, 1.0] | 0.77 | 5.36E-06 |
| 40 | PCaeC38:3* | 0.82 [0.57, 1.0] | 0.67 | 9.86E-06 |
| 41 | lysoPCaC28:0* | 0.82 [0.60, 1.0] | 0.78 | 8.96E-06 |
| 42 | SM (OH) C22:2* | 0.82 [0.58, 1.0] | 0.73 | 1.05E-05 |
| 43 | PCaeC36:4* | 0.81 [0.60, 1.0] | 0.75 | 1.48E-05 |
| 44 | PCaeC42:2* | 0.81 [0.53, 1.0] | 0.68 | 2.08E-05 |
| 45 | PCaaC30:0* | 0.80 [0.56, 1.0] | 0.61 | 3.36E-05 |
| 46 | PCaaC36:5* | 0.80 [0.56, 1.0] | 0.56 | 2.42E-05 |
| 47 | PCaaC38:1 | 0.80 [0.47, 0.98] | 0.63 | 1.89E-05 |
| **Fig. 4B (COPD vs. Ctrl)** | | | | |
| **Rank** | **Lipid** | **Mean AUC [95% CI]** | **Ratio of medians** | **p-value** |
| 1 | PCaaC42:6* | 0.93 [0.67, 1.0] | 0.62 | 5.32E-06 |
| 2 | PCaaC42:4* | 0.82 [0.51, 1.0] | 0.79 | 4.92E-04 |
| 3 | PCaeC36:3 | 0.82 [0.43, 1.0] | 0.75 | 4.15E-04 |
| 4 | PCaaC42:5* | 0.82 [0.57, 1.0] | 0.78 | 7.24E-04 |
| 5 | PCaeC34:3 | 0.81 [0.38, 1.0] | 0.67 | 6.12E-04 |
| 6 | lysoPCaC24:0 | 0.80 [0.43, 1.0] | 0.76 | 8.23E-04 |
| 7 | PCaaC38:5* | 0.80 [0.50, 1.0] | 0.71 | 1.35E-03 |
| 8 | PCaeC38:0 | 0.80 [0.38, 1.0] | 0.56 | 1.24E-03 |
| 9 | PCaaC40:3* | 0.80 [0.50, 1.0] | 0.79 | 1.10E-03 |
| **Fig. 4C (CAP vs. COPD)** | | | | |
| **Rank** | **Lipid** | **Mean AUC [95% CI]** | **Ratio of medians** | **p-value** |
| 1 | lysoPCaC20:4* | 0.83 [0.56, 1.0] | 0.62 | 3.19E-04 |
| 2 | PCaeC40:1 | 0.83 [0.40, 1.0] | 0.66 | 3.04E-04 |
| 3 | PCaaC34:4 | 0.82 [0.39, 1.0] | 0.52 | 3.36E-04 |
| 4 | PCaaC28:1* | 0.82 [0.50, 1.0] | 0.7 | 4.08E-04 |
| 5 | PCaeC38:5 | 0.82 [0.44, 1.0] | 0.75 | 4.72E-04 |
| 6 | SM C16:0* | 0.82 [0.50, 1.0] | 0.83 | 6.03E-04 |
| 7 | PCaaC32:3 | 0.82 [0.40, 1.0] | 0.72 | 4.97E-04 |
| 8 | SM C16:1* | 0.81 [0.50, 1.0] | 0.71 | 7.99E-04 |
| 9 | lysoPCaC18:2 | 0.81 [0.44, 1.0] | 0.46 | 5.48E-04 |
| 10 | lysoPCaC16:1 | 0.80 [0.30, 1.0] | 0.48 | 8.37E-04 |
| 11 | lysoPCaC18:1 | 0.80 [0.44, 1.0] | 0.58 | 8.37E-04 |
| 12 | lysoPCaC16:0 | 0.80 [0.33, 1.0] | 0.67 | 1.01E-03 |
| **Fig. 4D (CAP vs. Ctrl)** | | | | |
| **Rank** | **Indicator** | **Mean AUC [95% CI]** | **Ratio of medians** | **p-value** |
| 1 | Total lysoPC* | 0.93 [0.71, 1.0] | 0.56 | 4.87E-09 |
| 2 | Total lysoPC / Total PC* | 0.88 [0.62, 1.0] | 0.75 | 1.20E-07 |
| 3 | Total PC ae* | 0.88 [0.64, 1.0] | 0.71 | 1.96E-07 |
| 4 | PUFA (PC)* | 0.87 [0.69, 1.0] | 0.80 | 3.29E-07 |
| 5 | Total PC* | 0.82 [0.64, 1.0] | 0.82 | 9.57E-06 |
| 6 | Total (PC+SM)* | 0.81 [0.64, 1.0] | 0.81 | 1.16E-05 |
| 7 | Total SM-OH / Total SM-non OH* | 0.80 [0.53, 1.0] | 0.80 | 2.26E-05 |
| 8 | PUFA (PC) / SFA (PC)* | 0.80 [0.57, 1.0] | 0.84 | 1.90E-05 |
| 9 | Total PC aa* | 0.80 [0.60, 1.0] | 0.82 | 3.68E-05 |
| **Fig. 4E (COPD vs. Ctrl)** | | | | |
| **Rank** | **Indicator** | **Mean AUC [95% CI]** | **Ratio of medians** | **p-value** |
| 1 | Total SM / Total PC | 0.86 [0.42, 1.0] | 1.29 | 8.09E-05 |
| 2 | Total SM / Total (SM+PC) | 0.85 [0.42, 1.0] | 1.29 | 9.83E-05 |
| 3 | lysoPC a C20:4 / lysoPC a C20:3 | 0.85 [0.48, 1.0] | 1.21 | 1.76E-04 |
| 4 | Total SM-OH / Total SM-non OH* | 0.81 [0.50, 1.0] | 0.82 | 9.68E-04 |
| 5 | PUFA (PC) / SFA (PC)* | 0.80 [0.50, 1.0] | 0.89 | 8.24E-04 |
| **Fig. 4F (CAP vs. COPD)** | | | | |
| **Rank** | **Indicator** | **Mean AUC [95% CI]** | **Ratio of medians** | **p-value** |
| 1 | Total SM* | 0.81 [0.50, 1.0] | 0.79 | 9.60E-04 |
| 2 | Total lysoPC | 0.80 [0.40, 1.0] | 0.66 | 7.62E-04 |

| **Table S4:** Differences in biomarker potential among the lipid classes | | | | |
| --- | --- | --- | --- | --- |
| **Lipid class** | **No. included in analysis** | **No. qualifying as biomarker ^a,b^** | **% of all biomarkers** | **% of class** |
| All | 111 | 55 | 100 | n/a |
| PC | 70 | 39 | 71 | 47 |
| lysoPC | 13 | 11 | 20 | 77 |
| SM | 14 | 5 | 9 | 21 |
| AC | 14 | 0 | 0 | 0 |
| ^a^ AUC ≥ 0.8, lower bound AUC 95%CI ≥ 0.5, and p < 0.05.  ^b^ If the same analyte qualified as biomarker in 2 comparisons, it was counted as 2. | | | | |

| **Table S5: Effect of modelling increasing the sample size of COPD to that of CAP (n=29) by an over-sampling approach.** The symbol * in the Lipid/Indicator column indicates accurate biomarkers as defined by AUC ≥ 0.8, asymptotic p < 0.05 and AUC lower bound 95% CI ≥ 0.5. The number of accurate biomarkers for COPD/Ctrl now increased from 5 to 13. | | | | |
| --- | --- | --- | --- | --- |
| **Rank** | **Lipid** | **Mean AUC [95% CI]** | **Ratio of medians** | **p-value** |
| 1 | PCaaC42:6* | 0.94 [0.81, 1.0] | 0.62 | 2.35E-09 |
| 2 | PCaeC36:3* | 0.86 [0.57, 1.0] | 0.73 | 4.20E-07 |
| 3 | PCaaC42:4* | 0.86 [0.67, 1.0] | 0.78 | 4.97E-07 |
| 4 | PCaeC34:3* | 0.84 [0.60, 1.0] | 0.57 | 1.89E-06 |
| 5 | PCaaC40:3* | 0.83 [0.61, 1.0] | 0.77 | 3.14E-06 |
| 6 | lysoPCaC18:0* | 0.82 [0.56, 1.0] | 0.58 | 8.90E-06 |
| 7 | PCaeC38:2* | 0.81 [0.55, 1.0] | 0.74 | 1.18E-05 |
| 8 | PCaaC42:2* | 0.81 [0.57, 1.0] | 0.70 | 1.82E-05 |
| 9 | PCaaC42:5* | 0.81 [0.54, 1.0] | 0.78 | 9.45E-06 |
| 10 | lysoPCaC24:0* | 0.80 [0.57, 1.0] | 0.76 | 2.55E-05 |
| 11 | PCaeC38:0* | 0.80 [0.54, 1.0] | 0.54 | 2.71E-05 |
| 12 | PCaaC36:2* | 0.80 [0.50, 1.0] | 0.85 | 2.55E-05 |
| 13 | lysoPCaC28:0* | 0.80 [0.53, 1.0] | 0.88 | 2.87E-05 |

| **Table S6 (to Fig. 5A,B):** Identification of plasma lipids and metabolic indicators that correlate with resolution of inflammation and clinical improvement. | | | |
| --- | --- | --- | --- |
| **Fig. 5A** | | | |
| **Rank** | **Lipid** | **LFS** | **(1 - NDF)** |
| 1 | lysoPCaC16:1 | 0.156 | 0.946 |
| 2 | lysoPCaC16:0 | 0.124 | 0.940 |
| 3 | lysoPCaC17:0 | 0.122 | 0.947 |
| 4 | lysoPCaC18:0 | 0.122 | 0.937 |
| 5 | lysoPCaC20:4 | 0.120 | 0.905 |
| 6 | lysoPCaC20:3 | 0.118 | 0.915 |
| 7 | PCaeC40:1 | 0.116 | 0.884 |
| 8 | lysoPCaC18:1 | 0.107 | 0.940 |
| 9 | lysoPCaC18:2 | 0.106 | 0.968 |
| 10 | PCaaC34:4 | 0.102 | 0.852 |
| 11 | PCaeC44:6 | 0.088 | 0.865 |
| 12 | PCaaC38:3 | 0.084 | 0.899 |
| 13 | PCaaC36:6 | 0.082 | 0.880 |
| 14 | PCaeC42:5 | 0.081 | 0.914 |
| 15 | PCaeC36:5 | 0.077 | 0.890 |
| 16 | C6C41DC | 0.075 | 0.910 |
| 17 | PCaeC40:3 | 0.072 | 0.898 |
| 18 | PCaeC36:4 | 0.072 | 0.883 |
| 19 | PCaeC40:4 | 0.070 | 0.897 |
| 20 | PCaaC36:0 | 0.068 | 0.868 |
| 21 | C18:1 | 0.066 | 0.853 |
| 22 | PCaeC38:5 | 0.066 | 0.885 |
| 23 | PCaeC38:3 | 0.064 | 0.903 |
| 24 | PCaaC36:3 | 0.063 | 0.886 |
| 25 | PCaeC40:5 | 0.063 | 0.851 |
| **Fig. 5B** | | | |
| **Rank** | **Indicator** | **LFS** | **(1 - NDF)** |
| 1 | Total lysoPC | 0.110 | 0.937 |
| 2 | lysoPCaC16:0 / lysoPCaC16:1 | 0.095 | 0.877 |
| Abbreviations: LFS = linear fitting slope; NDF = normalized distance to fitting | | | |

| **Table S7 (to Fig. 7A, E and G):** Correlation of plasma lipids and metabolic indicators with CRP and PCT as measures of systemic inflammation. Sorted by absolute correlation coefficient, ccoef (CRP). | | | | | |
| --- | --- | --- | --- | --- | --- |
| **Fig. 7A** | | | | | |
| **Rank** | **Lipid** | **ccoef (CRP)** | **p-value** | **ccoef (PCT)** | **p-value** |
| 1 | PCaeC40:1 | -0.68 | 1.05E-17 | -0.36 | 4.33E-05 |
| 2 | lysoPCaC16:0 | -0.65 | 4.83E-16 | -0.37 | 2.16E-05 |
| 3 | lysoPCaC17:0 | -0.65 | 2.49E-16 | -0.37 | 1.97E-05 |
| 4 | lysoPCaC18:1 | -0.55 | 5.41E-11 | -0.30 | 8.86E-04 |
| 5 | lysoPCaC16:1 | -0.53 | 4.02E-10 | -0.24 | 6.61E-03 |
| 6 | lysoPCaC18:0 | -0.53 | 2.58E-10 | -0.34 | 1.31E-04 |
| 7 | PCaeC38:4 | -0.52 | 1.08E-09 | -0.41 | 2.18E-06 |
| 8 | PCaeC38:5 | -0.52 | 5.51E-10 | -0.43 | 5.27E-07 |
| 9 | lysoPCaC18:2 | -0.51 | 1.44E-09 | -0.27 | 2.80E-03 |
| 10 | lysoPCaC20:4 | -0.51 | 1.58E-09 | -0.34 | 1.18E-04 |
| 11 | PCaaC34:4 | -0.51 | 1.75E-09 | -0.20 | 2.49E-02 |
| 12 | PCaeC38:3 | -0.51 | 1.21E-09 | -0.35 | 7.88E-05 |
| 13 | PCaeC40:5 | -0.51 | 2.26E-09 | -0.42 | 1.52E-06 |
| 14 | PCaeC42:3 | -0.51 | 1.46E-09 | -0.34 | 1.52E-04 |
| 15 | PCaeC42:2 | -0.50 | 4.24E-09 | -0.32 | 3.79E-04 |
| 16 | PCaeC36:4 | -0.49 | 9.78E-09 | -0.34 | 1.39E-04 |
| 17 | lysoPCaC20:3 | -0.48 | 2.35E-08 | -0.25 | 5.59E-03 |
| 18 | PCaeC36:3 | -0.46 | 7.15E-08 | -0.29 | 9.64E-04 |
| 19 | PCaeC38:0 | -0.46 | 6.04E-08 | -0.19 | 3.27E-02 |
| 20 | PCaeC38:6 | -0.46 | 1.18E-07 | -0.30 | 6.67E-04 |
| 21 | PCaaC36:3 | -0.45 | 2.37E-07 | -0.21 | 2.16E-02 |
| 22 | PCaeC36:5 | -0.45 | 1.50E-07 | -0.36 | 5.07E-05 |
| 23 | PCaeC44:5 | -0.45 | 2.26E-07 | -0.34 | 1.08E-04 |
| 24 | PCaaC32:3 | -0.44 | 4.55E-07 | -0.29 | 1.15E-03 |
| 25 | PCaeC40:4 | -0.44 | 2.97E-07 | -0.35 | 7.40E-05 |
| 26 | PCaeC40:6 | -0.44 | 4.37E-07 | -0.27 | 2.32E-03 |
| 27 | lysoPCaC24:0 | -0.43 | 7.60E-07 | -0.27 | 2.11E-03 |
| 28 | PCaeC38:2 | -0.43 | 7.96E-07 | -0.28 | 1.44E-03 |
| 29 | PCaaC42:4 | -0.42 | 1.34E-06 | -0.30 | 6.49E-04 |
| 30 | PCaaC42:6 | -0.42 | 1.52E-06 | -0.23 | 9.06E-03 |
| 31 | PCaaC36:6 | -0.41 | 2.26E-06 | -0.13 | 1.44E-01 |
| 32 | PCaaC34:3 | -0.40 | 6.03E-06 | -0.10 | 2.76E-01 |
| 33 | PCaeC34:2 | -0.40 | 4.49E-06 | -0.22 | 1.50E-02 |
| 34 | PCaeC42:4 | -0.40 | 5.02E-06 | -0.30 | 7.93E-04 |
| 35 | PCaaC38:5 | -0.39 | 9.71E-06 | -0.19 | 3.67E-02 |
| 36 | PCaaC38:3 | -0.38 | 1.19E-05 | -0.25 | 6.04E-03 |
| 37 | PCaaC42:0 | -0.38 | 1.25E-05 | -0.26 | 3.50E-03 |
| 38 | C16 | -0.36 | 5.52E-05 | -0.18 | 4.36E-02 |
| 39 | PCaaC40:2 | -0.36 | 5.33E-05 | -0.25 | 4.62E-03 |
| 40 | PCaeC40:3 | -0.36 | 4.11E-05 | -0.37 | 3.01E-05 |
| 41 | PCaeC44:4 | -0.36 | 5.41E-05 | -0.25 | 4.67E-03 |
| 42 | PCaaC38:0 | -0.35 | 7.60E-05 | -0.26 | 3.49E-03 |
| 43 | PCaeC34:3 | -0.35 | 7.72E-05 | -0.24 | 6.83E-03 |
| 44 | PCaeC36:2 | -0.35 | 5.85E-05 | -0.19 | 3.98E-02 |
| 45 | PCaeC44:6 | -0.35 | 6.33E-05 | -0.31 | 4.02E-04 |
| 46 | SM (OH) C22:2 | -0.35 | 6.70E-05 | -0.32 | 2.65E-04 |
| 47 | PCaaC38:1 | -0.34 | 1.12E-04 | -0.21 | 2.09E-02 |
| 48 | PCaaC40:4 | -0.34 | 1.38E-04 | -0.27 | 2.90E-03 |
| 49 | lysoPCaC28:1 | -0.33 | 1.65E-04 | -0.17 | 6.78E-02 |
| 50 | PCaaC42:2 | -0.33 | 2.41E-04 | -0.22 | 1.26E-02 |
| 51 | SM (OH) C22:1 | -0.33 | 2.09E-04 | -0.33 | 1.79E-04 |
| 52 | PCaaC32:2 | -0.32 | 3.25E-04 | -0.03 | 7.64E-01 |
| 53 | PCaaC36:0 | -0.32 | 2.90E-04 | -0.2 | 2.37E-02 |
| 54 | PCaaC42:1 | -0.32 | 3.55E-04 | -0.18 | 5.04E-02 |
| 55 | PCaeC42:5 | -0.32 | 2.54E-04 | -0.07 | 4.10E-01 |
| 56 | PCaaC32:0 | 0.31 | 5.23E-04 | 0.37 | 2.08E-05 |
| 57 | lysoPCaC26:1 | -0.30 | 6.22E-04 | -0.21 | 1.95E-02 |
| 58 | PCaaC40:3 | -0.30 | 6.43E-04 | -0.27 | 2.36E-03 |
| 59 | PCaaC28:1 | -0.29 | 1.29E-03 | -0.12 | 1.91E-01 |
| 60 | PCaaC42:5 | -0.29 | 1.03E-03 | -0.23 | 1.05E-02 |
| 61 | PCaeC30:0 | -0.29 | 1.14E-03 | -0.09 | 3.14E-01 |
| 62 | PCaeC32:1 | -0.29 | 1.07E-03 | -0.18 | 4.60E-02 |
| 63 | PCaeC32:2 | -0.29 | 1.20E-03 | -0.20 | 2.32E-02 |
| 64 | PCaaC40:5 | -0.27 | 2.38E-03 | -0.17 | 6.70E-02 |
| 65 | PCaeC30:2 | -0.27 | 2.35E-03 | -0.17 | 5.79E-02 |
| 66 | PCaeC40:2 | -0.27 | 2.74E-03 | -0.26 | 3.70E-03 |
| 67 | PCaaC36:1 | -0.26 | 3.46E-03 | -0.10 | 2.92E-01 |
| 68 | PCaeC36:1 | -0.26 | 3.26E-03 | -0.09 | 3.26E-01 |
| 69 | SM C24:0 | -0.26 | 3.07E-03 | -0.29 | 9.78E-04 |
| 70 | C18 | -0.25 | 5.74E-03 | -0.14 | 1.21E-01 |
| 71 | SM C16:0 | -0.24 | 7.63E-03 | -0.29 | 1.03E-03 |
| 72 | PCaaC30:0 | -0.23 | 1.03E-02 | 0.03 | 7.26E-01 |
| 73 | SM (OH) C16:1 | -0.23 | 1.00E-02 | -0.25 | 5.97E-03 |
| 74 | C18:1 | 0.22 | 1.58E-02 | 0.20 | 2.54E-02 |
| 75 | PCaaC36:4 | -0.22 | 1.38E-02 | -0.19 | 3.36E-02 |
| 76 | PCaaC36:5 | -0.22 | 1.48E-02 | -0.06 | 5.41E-01 |
| 77 | lysoPCaC26:0 | -0.21 | 1.93E-02 | -0.18 | 4.41E-02 |
| 78 | SM (OH) C14:1 | -0.21 | 2.15E-02 | -0.16 | 8.41E-02 |
| 79 | SM C24:1 | -0.21 | 1.87E-02 | -0.33 | 2.15E-04 |
| 80 | PCaeC36:0 | 0.20 | 2.69E-02 | 0.17 | 6.27E-02 |
| 81 | SM C18:1 | -0.20 | 2.56E-02 | -0.25 | 4.46E-03 |
| 82 | lysoPCaC28:0 | -0.19 | 3.85E-02 | -0.21 | 2.07E-02 |
| 83 | PCaaC38:6 | -0.18 | 4.38E-02 | 0.06 | 5.11E-01 |
| 84 | PCaaC32:1 | -0.17 | 5.37E-02 | 0.09 | 2.99E-01 |
| 85 | SM C18:0 | -0.17 | 6.08E-02 | -0.28 | 1.61E-03 |
| 86 | C14:1 | -0.16 | 7.23E-02 | -0.17 | 5.59E-02 |
| 87 | SM C26:0 | -0.16 | 8.28E-02 | -0.22 | 1.58E-02 |
| 88 | PCaaC38:4 | -0.15 | 1.08E-01 | -0.24 | 8.10E-03 |
| 89 | PCaeC34:0 | -0.15 | 9.61E-02 | -0.02 | 8.01E-01 |
| 90 | PCaeC34:1 | -0.15 | 9.30E-02 | -0.02 | 7.84E-01 |
| 91 | SM C16:1 | -0.15 | 1.01E-01 | -0.12 | 1.72E-01 |
| 92 | C0 | 0.13 | 1.55E-01 | 0.28 | 1.73E-03 |
| 93 | PCaaC36:2 | -0.12 | 1.90E-01 | -0.06 | 5.12E-01 |
| 94 | SM C20:2 | 0.12 | 1.73E-01 | -0.14 | 1.24E-01 |
| 95 | C4 | 0.11 | 2.33E-01 | 0.21 | 1.71E-02 |
| 96 | SM C26:1 | -0.11 | 2.32E-01 | -0.25 | 4.94E-03 |
| 97 | C10 | 0.09 | 3.00E-01 | 0.00 | 9.82E-01 |
| 98 | C18:2 | -0.09 | 3.46E-01 | -0.10 | 2.68E-01 |
| 99 | C3DCC4OH | 0.08 | 3.75E-01 | -0.10 | 2.58E-01 |
| 100 | PCaaC24:0 | 0.08 | 3.80E-01 | 0.02 | 7.86E-01 |
| 101 | PCaaC40:6 | -0.08 | 3.83E-01 | 0.03 | 7.32E-01 |
| 102 | C3 | 0.07 | 4.24E-01 | 0.13 | 1.51E-01 |
| 103 | PCaeC44:3 | -0.07 | 4.62E-01 | -0.04 | 6.91E-01 |
| 104 | SM (OH) C24:1 | -0.06 | 5.18E-01 | -0.20 | 2.58E-02 |
| 105 | C5 | 0.05 | 5.83E-01 | -0.01 | 8.83E-01 |
| 106 | C2 | 0.04 | 6.74E-01 | 0.19 | 3.64E-02 |
| 107 | PCaaC34:1 | -0.04 | 6.90E-01 | 0.17 | 5.85E-02 |
| 108 | PCaeC38:1 | -0.01 | 8.70E-01 | -0.01 | 9.29E-01 |
| 109 | C6C41DC | 0.00 | 9.65E-01 | -0.17 | 6.47E-02 |
| 110 | C8 | 0.00 | 9.75E-01 | -0.02 | 8.56E-01 |
| 111 | PCaaC34:2 | 0.00 | 9.67E-01 | 0.14 | 1.22E-01 |
| **Fig. 7E** | | | | | |
| **Rank** | **Ceramides** | **ccoef (CRP)** | **p-value** | **ccoef (PCT)** | **p-value** |
| 1 | Cer 24:0 | 0.24 | 7.81E-03 | 0.36 | 3.44E-05 |
| 2 | Cer 18:0 | 0.15 | 1.02E-01 | 0.05 | 5.84E-01 |
| 3 | Cer 16:0 | 0.12 | 1.85E-01 | 0.05 | 6.19E-01 |
| 4 | Cer 24:1 | 0.09 | 3.29E-01 | 0.07 | 4.23E-01 |
| **Fig. 7G** | | | | | |
| **Rank** | **Indicator** | **ccoef (CRP)** | **p-value** | **ccoef (PCT)** | **p-value** |
| 1 | Total lysoPC / Total PC | -0.64 | 2.29E-15 | -0.42 | 9.63E-07 |
| 2 | Total lysoPC | -0.63 | 8.55E-15 | -0.36 | 4.96E-05 |
| 3 | Total PC ae | -0.51 | 1.32E-09 | -0.34 | 1.00E-04 |
| 4 | lysoPCaC16:0 / lysoPCaC16:1 | 0.39 | 7.81E-06 | 0.03 | 7.12E-01 |
| 5 | PUFA (PC) / SFA (PC) | -0.39 | 7.45E-06 | -0.35 | 6.67E-05 |
| 6 | PCaeC44:5 / PCaeC42:5 | -0.35 | 6.73E-05 | -0.32 | 2.71E-04 |
| 7 | C16 / C16:1 | -0.34 | 9.83E-05 | -0.04 | 6.34E-01 |
| 8 | C18 / C18:1 | -0.33 | 2.27E-04 | -0.14 | 1.23E-01 |
| 9 | PCaaC36:3 / PCaaC36:4 | -0.30 | 7.23E-04 | -0.01 | 8.88E-01 |
| 10 | Total SM-OH | -0.29 | 1.09E-03 | -0.27 | 2.21E-03 |
| 11 | C9 / C14 | 0.26 | 3.59E-03 | 0.20 | 2.79E-02 |
| 12 | PUFA (PC) | -0.26 | 4.05E-03 | -0.09 | 2.97E-01 |
| 13 | Total SM | -0.26 | 4.23E-03 | -0.32 | 3.28E-04 |
| 14 | PCaaC28:1 / PCaaC38:1 | 0.25 | 4.82E-03 | 0.21 | 2.28E-02 |
| 15 | Total SM-non OH | -0.24 | 7.34E-03 | -0.31 | 4.22E-04 |
| 16 | PCaeC32:1 / PCaeC34:1 | -0.23 | 1.15E-02 | -0.19 | 3.27E-02 |
| 17 | MUFA (PC) / SFA (PC) | -0.22 | 1.46E-02 | -0.05 | 5.52E-01 |
| 18 | Total (PC+SM) | -0.22 | 1.24E-02 | -0.04 | 6.22E-01 |
| 19 | Total PC | -0.22 | 1.57E-02 | -0.03 | 7.44E-01 |
| 20 | C12 / C8 | -0.21 | 1.86E-02 | -0.29 | 1.12E-03 |
| 21 | lysoPCaC20:4 / lysoPCaC20:3 | 0.21 | 2.14E-02 | -0.14 | 1.20E-01 |
| 22 | Total SM-OH / Total SM-non OH | -0.21 | 1.86E-02 | -0.14 | 1.25E-01 |
| 23 | C12 / C10 | -0.19 | 3.93E-02 | -0.16 | 7.30E-02 |
| 24 | Total PC aa | -0.19 | 3.91E-02 | 0.00 | 9.64E-01 |
| 25 | CPT-I ratio | -0.18 | 4.91E-02 | -0.16 | 6.88E-02 |
| 26 | PUFA (PC) / MUFA (PC) | -0.18 | 4.62E-02 | -0.30 | 7.21E-04 |
| 27 | Total AC-OH / Total AC | 0.18 | 4.91E-02 | 0.02 | 8.50E-01 |
| 28 | C9 / C10:2 | -0.17 | 6.36E-02 | -0.07 | 4.51E-01 |
| 29 | PCaaC40:3 / PCaaC42:5 | 0.14 | 1.27E-01 | 0.09 | 3.04E-01 |
| 30 | PCaaC28:1 / PCaaC40:2 | 0.13 | 1.60E-01 | 0.22 | 1.44E-02 |
| 31 | C14:1-OH / C10 | -0.12 | 1.89E-01 | -0.12 | 1.74E-01 |
| 32 | C12 / C12:1 | -0.11 | 2.20E-01 | -0.08 | 3.90E-01 |
| 33 | MUFA (PC) | -0.10 | 2.75E-01 | 0.12 | 1.76E-01 |
| 34 | Total AC-DC / Total AC | -0.10 | 2.61E-01 | -0.18 | 4.28E-02 |
| 35 | SFA (PC) | 0.09 | 3.14E-01 | 0.22 | 1.55E-02 |
| 36 | C14 / C16:1 | -0.07 | 4.61E-01 | 0.02 | 8.00E-01 |
| 37 | C4 / C0 | 0.07 | 4.60E-01 | 0.09 | 3.29E-01 |
| 38 | Total SM / Total (SM+PC) | -0.07 | 4.74E-01 | -0.33 | 1.76E-04 |
| 39 | Total SM / Total PC | -0.06 | 4.94E-01 | -0.32 | 2.50E-04 |
| 40 | C9 / C14:1-OH | -0.04 | 6.35E-01 | 00.0 | 9.65E-01 |
| 41 | C9 / C16:1-OH | -0.04 | 6.49E-01 | 00.0 | 9.82E-01 |
| 42 | C4 / C5 | -0.03 | 7.40E-01 | 0.20 | 2.38E-02 |
| 43 | Total AC / C0 | 0.03 | 7.42E-01 | -0.01 | 9.54E-01 |
| 44 | C3 / C4 | -0.02 | 8.25E-01 | -0.05 | 5.48E-01 |
| 45 | PCaaC40:5 / PCaaC42:5 | -0.02 | 8.58E-01 | 0.07 | 4.14E-01 |
| 46 | C2 / C0 | -0.01 | 9.51E-01 | 0.04 | 6.98E-01 |
| 47 | (C2+C3) / C0 | 0.00 | 9.60E-01 | 0.03 | 7.02E-01 |
